# Atomistic description of the OCTN1 recognition mechanism via in silico methods

**DOI:** 10.1371/journal.pone.0304512

**Published:** 2024-06-03

**Authors:** Omar Ben Mariem, Luca Palazzolo, Beatrice Torre, Yao Wei, Davide Bianchi, Uliano Guerrini, Tommaso Laurenzi, Simona Saporiti, Emma De Fabiani, Lorena Pochini, Cesare Indiveri, Ivano Eberini

**Affiliations:** 1 Dipartimento di Scienze Farmacologiche e Biomolecolari, Università degli Studi di Milano, Milan, Italy; 2 Analytical Excellence and Program Management, Merck Serono S.p.A., Rome, Italy; 3 Dipartimento di Biologia, Ecologia e Scienze della Terra, Università della Calabria, Arcavacata CS, Italy; 4 CNR Institute of Biomembranes, Bioenergetics and Molecular Biotechnologies (IBIOM), Bari, Italy; 5 DSRC, Università degli Studi di Milano, Milan, Italy; Cukurova University: Cukurova Universitesi, TURKEY

## Abstract

The Organic Cation Transporter Novel 1 (OCTN1), also known as SLC22A4, is widely expressed in various human tissues, and involved in numerous physiological and pathological processes remains. It facilitates the transport of organic cations, zwitterions, with selectivity for positively charged solutes. Ergothioneine, an antioxidant compound, and acetylcholine (Ach) are among its substrates. Given the lack of experimentally solved structures of this protein, this study aimed at generating a reliable 3D model of OCTN1 to shed light on its substrate-binding preferences and the role of sodium in substrate recognition and transport. A chimeric model was built by grafting the large extracellular loop 1 (EL1) from an AlphaFold-generated model onto a homology model. Molecular dynamics simulations revealed domain-specific mobility, with EL1 exhibiting the highest impact on overall stability. Molecular docking simulations identified cytarabine and verapamil as highest affinity ligands, consistent with their known inhibitory effects on OCTN1. Furthermore, MM/GBSA analysis allowed the categorization of substrates into weak, good, and strong binders, with molecular weight strongly correlating with binding affinity to the recognition site. Key recognition residues, including Tyr211, Glu381, and Arg469, were identified through interaction analysis. Ach demonstrated a low interaction energy, supporting the hypothesis of its one-directional transport towards to outside of the membrane. Regarding the role of sodium, our model suggested the involvement of Glu381 in sodium binding. Molecular dynamics simulations of systems at increasing levels of Na+ concentrations revealed increased sodium occupancy around Glu381, supporting experimental data associating Na+ concentration to molecule transport. In conclusion, this study provides valuable insights into the 3D structure of OCTN1, its substrate-binding preferences, and the role of sodium in the recognition. These findings contribute to the understanding of OCTN1 involvement in various physiological and pathological processes and may have implications for drug development and disease management.

## Introduction

The human Organic Cation Transporter Novel 1 (OCTN1) is a protein encoded by the *SLC22A4* gene [1, 2] (UniProt ID: Q9H015). OCTN1 is a polyspecific transporter responsible for the transport of organic cations and zwitterions. Moreover, it is reported that OCTN1 can transport some substrates regardless of the presence of sodium while the transport of other molecules is sodium dependent [2, 3]. In humans, OCTN1 is widely expressed in the plasma membrane of different tissues, and it is involved in many physiological and pathological processes [4–13]. This transporter is present in immune cells, in the brush border of intestinal cells, in the lumen of renal proximal tubule cells, in tracheal cells, in the lungs and, albeit to a lesser extent, in the uterus, pancreas, heart, spleen, bone marrow and placenta [3].

Historically, it has been known as OCTN1 due to the wide variety of transported substrates, all presenting a positive charge [2, 14, 15]. However, in recent years, some research groups have been suggesting the adoption of the name Ergothioneine transporter (ETT) [2], due to its high affinity for this exogenous molecule. In this paper we will refer to this protein using the original acronym OCTN1 and we believe it is important to highlight the current controversy.

Ergothioneine is a histidine-derived sulfur compound absorbed *via* the diet. It has been shown to have antioxidant properties since it is able to neutralize hydroxyl radicals and inhibit the production of oxidizing species by metal ions such as Fe^2+^ or Cu^2+^. The deficiency of OCTN1 on the plasma membrane drastically reduces the intracellular supply of ergothioneine [16]. Nevertheless, not much is known about the specific mechanism of transport of this molecule, such as the key residues involved, but literature agrees on the dependency on the sodium ion.

Other substrates of OCTN1 so far described are tetraethylammonium (TEA) and acetylcholine (Ach). The former is a small molecule with a single positive charge on a nitrogen atom linked to four ethyl groups. The latter is a physiological cation that, besides its well-recognized role as a neurotransmitter, also plays an important role in the non-neuronal cholinergic system. Indeed, OCTN1 can mediate Ach export in non-neuronal tissues, where Ach can be synthesized due to the expression of choline acetyltransferase [4]. In this context, Ach exported from cells plays several biological roles, such as in the cholinergic anti-inflammatory pathway, thanks to the interaction with two receptor subtypes mAChRs and nAChRs [17].

Among the endogenous compounds that were studied as possible OCTN1 substrates, carnitine, acetylcarnitine, and choline were also considered. Specifically, it was observed that carnitine can be transported by OCTN1, but with a lower affinity than that found in the case of OCTN2 towards the same compound. At the same time, carnitine inhibits TEA uptake in a non-competitive way, suggesting the existence of different binding sites [10, 12]. In particular, the presence of three subsites was hypothesized [3].

The main hypothesis concerning Ach is that OCTN1 is mainly involved in its efflux and that its transport is competitively inhibited by TEA and acetylcarnitine [3, 10, 18].

Furthermore, it appears that OCTN1 is a spermine carrier although it is not the only transporter involved in its absorption [19]. In addition to endogenous compounds, the transport of various drugs such as ipratropium [20], gabapentin [21], ethambutol [22], entecavir [23], sulpiride [24], and emtricitabine [25] was studied. These are compounds with at least one positive charge at physiological pH, but that belong to very different pharmacological classes. This supports the hypothesis that OCTN1 is involved in the pharmacokinetics of these molecules, in the processes of absorption and excretion, as well as in the development of side effects due to the intracellular accumulation of the active compound.

A peculiar behaviour was observed in the case of cytarabine (Ara-C) [26], an antitumor agent analogous to cytidine, since its transport is not inhibited by either TEA or ET, suggesting the possible existence of a different binding site with respect to that used by the organic cations mentioned so far. Indeed, a high expression of OCTN1 is associated with a better outcome of chemotherapy treatment with Ara-C in the therapy against acute myeloid leukemia was recently observed [26].

The molecules that, on the other hand, were shown to have an inhibitory effect on the activity of OCTN1 are quinidine, pyrilamine and verapamil, the latter considered to be the most powerful inhibitor of this transporter [23, 24].

In Table 1 the properties of some of the transported substrates and inhibitors are reported. As previously mentioned, they vary widely, ranging from amino acids, such as ergothioneine, to nucleoside analogs, biguanides, and quaternary ammonium ions, as well as having a wide range of molecular weights. This information, along with the observed non-competitive behaviour among certain substrates, supports the hypothesized presence of multiple possible binding modes or sub-sites inside the transport funnel [3].

**Table 1:** List of known solutes and inhibitors of OCNT1 associated to their chemical class and molecular weight.

Molecule name	Class	Molecular weight (g/mol)
Ergothioneine	Amino acid derivative	229.3
Stachydrine	Amino acid derivative	143.2
Homostachydrine	Amino acid derivative	157.2
TEA	Quaternary ammonium	130.3
Acetylcholine	Quaternary ammonium	146.2
Carnitine	Quaternary ammonium	161.2
Choline	Quaternary ammonium	104.2
2-deoxycytidine	Nucleoside analog	227.2
Clofarabine	Nucleoside analog	303.7
Emtricitabine	Nucleoside analog	247.3
Ribavirin	Nucleoside analog	244.2
Cytarabine	Nucleoside analog	243.2
5-fluorouracil	Nucleoside analog	130.1
Fludarabine	Nucleoside analog	285.2
Gemcitabine	Nucleoside analog	263.2
Entecavir	Nucleoside analog	277.3
Buformin	Biguanide	157.2
Metformin	Biguanide	129.2
Phenformin	Biguanide	205.3
Ethambutol	Ethylenediamine derivative	204.3
Tiotropium	Tropane derivative	392.5
Ipratropium	Tropane derivative	332.5
Verapamil	Phenylbutilamine	454.6
Spermine	Polyamine	202.3
Amilsulpride	Benzamide	369.5
Gabapentin	GABA analog	171.2
Quindine	Alkaloid*	324.4
Saracatinib	Quinazoline	542.0

In the currently available literature, OCTN1 mutations related to the onset of pathologies were investigated. Several studies demonstrated that the L503F mutation is a susceptibility index for the onset of Crohn’s disease [9], a chronic, inflammatory disease that involves the entire gastrointestinal tract. The L503F mutation is part of the haplotype responsible for the onset of the disease; therefore, other conditions/mutations are needed for this to manifest.

In summary, OCTN1 is involved in the transport of molecules with different characteristics both in terms of structure and activity. The variety of possible substrates suggests that this transporter is involved in different physiological and/or pathological processes.

The goal of this study is the generation of a reliable 3D model of OCTN1 that could be used for the identification of key structural features supporting the recognition of inhibitors and transported solutes. The obtained data will also help in classifying the studied molecules as weak or strong binders/inhibitors. The role of sodium in solute recognition will also be analyzed. This information will then be useful to shed some light on its substrate recognition and transport mechanisms, aiding targeted drug development and enhancing our understanding of related diseases like Crohn’s.

## Materials and methods

### Modelling of OCTN1

The sequence of the human OCTN1 (UniProt ID: Q9H015) was retrieved from the UniProt knowledgebase database [27]. After a protein BLAST [19] search in the Protein Data Bank (PDB) [28] for a human OCTN1 homolog, the structure of the human organic cation transporter 3 (OCT3, transcribed from the gene SLC22A3) was identified as the closest protein with an experimentally-solved structure. There are three different structures of the human OCT3 transporter (PDB ID:7ZH0; 7ZH6; 7ZHA) [29] and the one in complex with OCT3 inhibitor corticosterone (PDB ID: 7ZH6) was chosen as template for the homology modelling procedure. This structure has a favorable recognition site due to the presence of corticosterone and it is in an outward-facing conformation. The homology modelling procedure was performed using Prime [30, 31], from the Schrodinger 2022–3 suite (D. E. Shaw Research, New York, NY; Schrӧdinger, New York, NY).

Because of a low sequence identity in the large extracellular loop EL1 (residues 42–141), as well as gaps in the template structure, the quality of the modelled loop was not satisfactory. To overcome this structural problem, AlphaFold (AF) [32] was used to generate another model of OCTN1. The final chimeric model was finally obtained by integrating the EL1 from the AF model onto the structure obtained *via* homology modelling.

### Equilibration and cluster extrapolation

Molecular dynamics (MD) simulation and frame clustering procedures were carried out with the Small-Molecule Drug Discovery Suite from the Schrӧdinger 2022–3.

The Desmond System Builder tool was used to place the apo-model of OCTN1 into a 1-palmitoyl-2-oleoyl-sn-glycero-3-phosphocholine (POPC) membrane bilayer. Protein orientation was set up according to the OPM server [33], which provides spatial arrangements of membrane proteins with respect to the hydrocarbon core of the lipid bilayer. The system was solvated with SPC water molecules in a box considering a 10 Å buffer. Sodium and chloride ions were added to both sides of the bilayer to reach a 0.15 M concentration and neutralize the system [34–36]. The system was energy-minimized to relax the assembly and remove clashes between protein, membrane, and solvent. The total number of atoms was 82424, and the box size were 84.77 Å x 74.48 Å x 132.96 Å.

To produce an equilibrated model of OCTN1, the system was submitted to three replicas of a 1000 ns (1 μs) MD simulation using the Desmond Molecular Dynamics tool [37]. Periodic boundary conditions (PBCs) and the following parameters were set: 300 K and Nose–Hoover thermostat for temperature coupling, 1 bar and Martyna–Tobias–Klein piston for pressure coupling, and 2 fs as the integration time step. Coordinates and velocities of each atom were saved every 0.5 ps. OPLS4 was used as a force field to parametrize the atoms for the MD simulations [38].

The root mean square deviation (RMSD), the root mean square fluctuation (RMSF) of the alpha carbons of OCTN1 and interactions between OCTN1 and the solutes were calculated via Python scripts using the Schrödinger analysis API.

The Desmond Trajectory Frame Clustering tool was used to cluster the equilibrated part of MD simulations to select the most representative frame (the centroid) for each cluster. Distances between clusters were computed from the RMSD matrix of alpha carbons with respect to the first frame of the MD simulation.

Solvent energy distribution density of water and ions in the transport channel was also computed using the MOE solvent analysis module (RISM-3D).

### Molecular docking

The minimized structure of the chimeric model was used as receptor for the molecular docking procedure of the best-known solutes and inhibitors of OCTN1. The structures of the small molecules were downloaded from PubChem and prepared using the LigPrep tool in the Schrodinger suite using the OPLS4 force field. Table 1 reports the chemical information of the tested ligands.

The molecular docking procedure was carried out with Schrӧdinger Glide Induced Fit Docking [39] in the “extra precision” (XP) mode to evaluate the ability of the tested ligands to bind the OCTN1 recognition site, keeping only the five top-scoring poses [40].

The receptor box was built using the residues from the translocation funnel, specifically focusing on those located in the upper part of the funnel. These residues were chosen because of their interaction with the OCT3 inhibitor in the template structure. Ligands were considered flexible in all docking procedures to allow for multiple binding conformations.

### Ligand::protein interactions

To study protein::ligand interactions, refine the docking poses, and verify the stability of the ligand placement, the top-scoring docking pose of each ligand was submitted to two replicas of 100 ns of MD simulations. The above-mentioned parameters were set for each MD simulation, using OPLS4 as forcefield. The resulting simulations were analyzed using the Schrodinger API to obtain the lists of interactions between each ligand and the protein, MM/GBSA energy values, the RMSF values of key residues for the interaction, and the RMSD values of the solutes with respect to OCTN1. These RMSD values do not only consider the variations in solute conformations, but also its movement in 3D space.

### Analysis of the role of the sodium ion in the transport funnel

Based on the literature evidence suggesting sodium-dependence in the transport of certain molecules, notably ergothioneine, the potential binding sites for a sodium ion within the transport funnel w investigated. To achieve this, firstly, three replicas with two different Na+ concentrations were run (500mM and 1mM), using the same previously discussed parameters. The Na+ occupancy was calculated using the Schrödinger analysis API and a purposefully made Python script. Additionally, the MOE solvent analysis module was used to assess the density functions of Na+ ions in proximity to residues located within the transport funnel. Then, a sodium ion was placed at the position with the highest density and molecular docking of the solutes was carried out using this modified system (OCTN1::Na^+^) as the receptor.

## Results and discussion

### Model generation, equilibration, and analysis

Because of the lack of experimentally solved structures of OCTN1, the generation of a 3D model was the first necessary step for our study. As there is no indication of the need for a multimeric complex for OCTN1 function, the functional protein was considered and studied as a monomer for the whole study [2]. Homology modelling is the most reliable method for model generation, whenever an experimental structure of a similar protein is available to be used as a template. Currently, the only homologous protein with a sufficient percentage of sequence identity and an experimentally solved structure is OCT3, as found by a BLAST search using the OCTN1 primary structure as query. Therefore, a homology modelling procedure was attempted using the experimentally-solved structure with the PDB ID 7ZH6 [21] as a template, in the outward-facing conformation. The selection of the template was based on the presence of a ligand in the 7ZH6 Cryo-EM structure, indicating that the recognition site is already capable of binding molecules. This feature would make the generated model more suitable for the subsequent molecular docking procedure.

**Fig 1:**
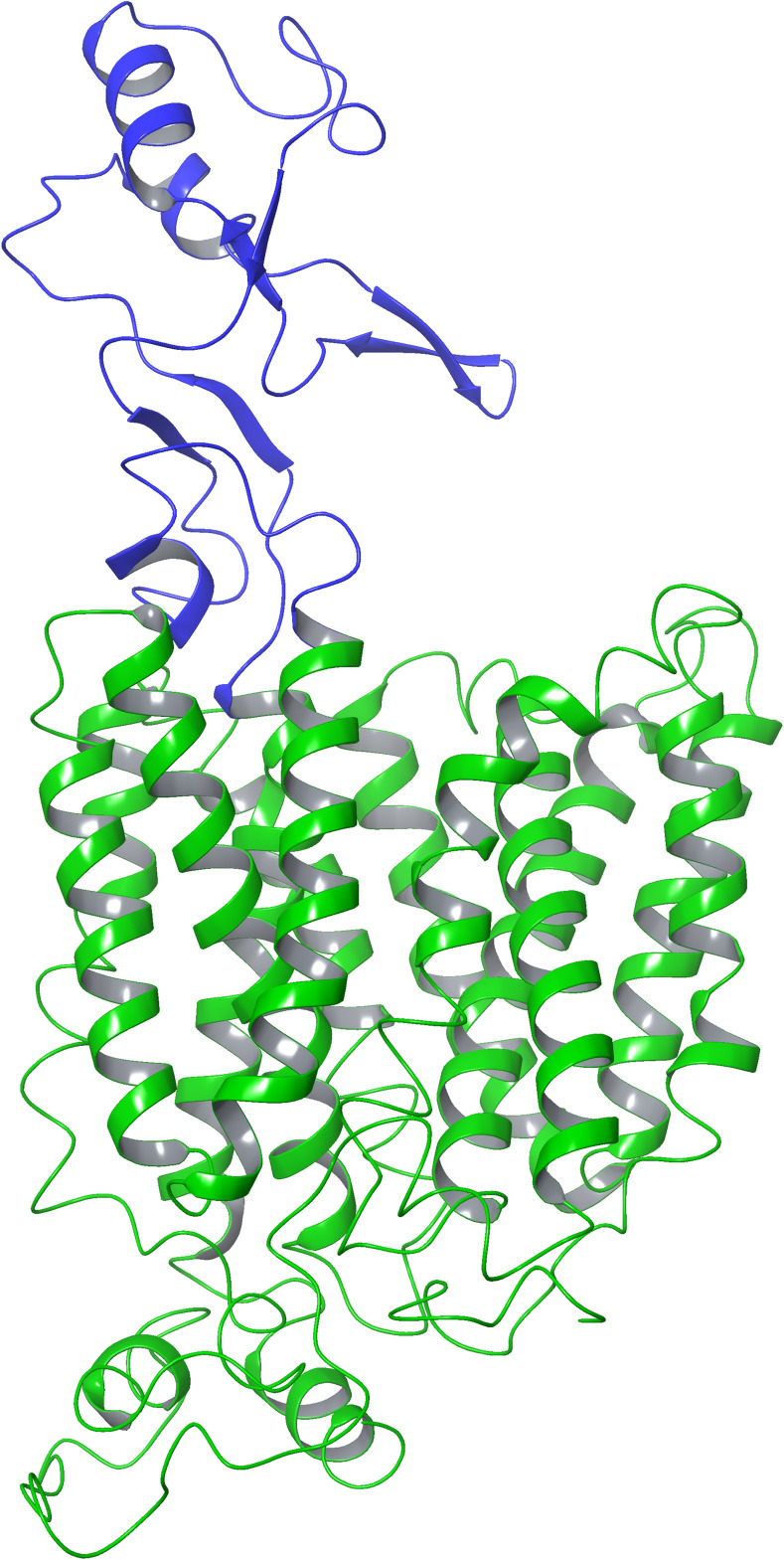
Chimeric model of OCNT1. In green, the portion obtained by homology modelling, in blue the EL1, obtained from AF. The extracellular portion is in the upper side of the figure.

Despite the satisfactory sequence identity in the transmembrane region (≈30%), the EL1 demonstrated a poor alignment (S1 Fig), as well as being poorly resolved in the OCT3 Cryo-EM (S2A–S2C Fig). Therefore, to obtain a better overall model, an attempt was made by generating the model of OCTN1 using AF. The model of OCTN1 already available in the AlphaFold database was compared to the newly generated one (S2D Fig), but no significant differences could be observed. Because the software is continuously updated and improved, the model generated with the newest version of the software was preferred. In particular, the AF model had a high overall confidence (S3 Fig), especially in the transmembrane region. However, it was not used as the definitive model for further calculations because of the lack of a clearly defined conformation (outward- or inward-facing), as seen in S4 Fig. Because of that, the EL1 of the AF model was grafted onto the transmembrane portion generated by homology modelling, obtaining a chimeric model with the best properties of both (Fig 1). Interestingly, in all obtained models, L503 was predicted to be on a transmembrane helix not facing the transport funnel. This might suggest that the role of the L503F mutation can lead to the development of Crohn’s syndrome by affecting the positioning in the membrane or the ability to change conformation of OCTN1, not its affinity for substrates.

**Fig 2:**
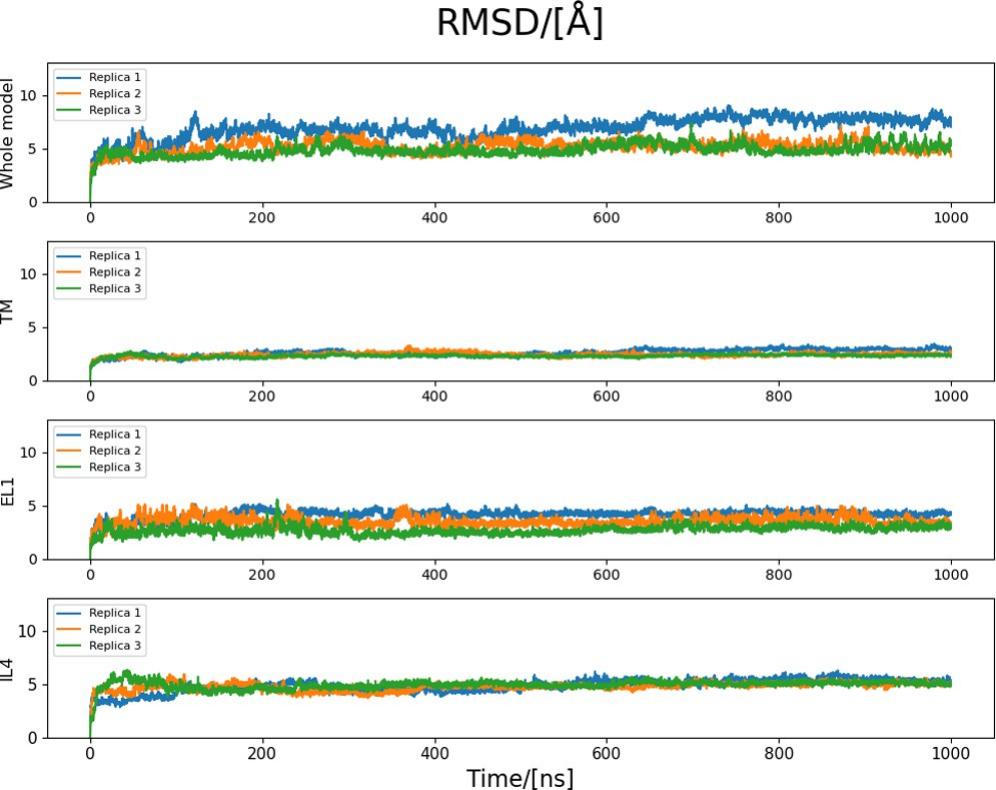
RMSD plots of the three MD replicas, considering the whole protein and a focus on each structural domain. It is possible to see how replicas reached a plateau rather quickly. The largest contributors to the high RMSD values are the EL1 and IL4, while the TM region maintains a constantly low RMSD profile.

**Fig 3:**
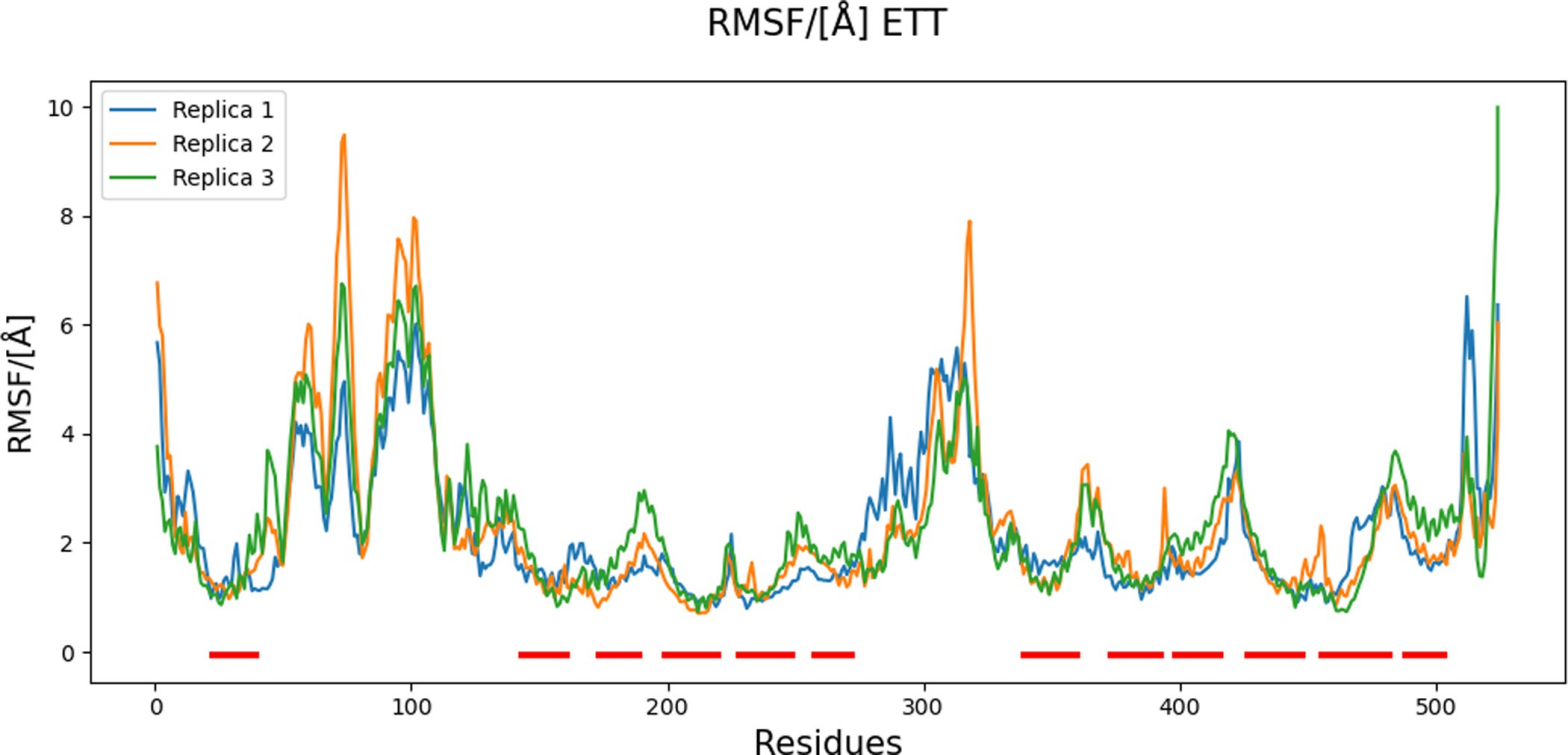
RMSF plot of the three MD replicas. Red horizontal lines on the bottom of the plot identify the transmembrane helices, on average, characterized by lower values of RMSF. The highest peaks characterize the EL1 and IL4.

Three replicas of molecular dynamics simulations were performed on the minimized chimeric model after inserting it in a POPC bilayer. The RMSD and RMSF values were calculated for the alpha carbons of the model during the simulations. Due to the high variability in mobility among the different portions of the protein, domain-specific analyses were performed. In particular, the EL1 and IL4 (residues 279–337) have the highest impact on the overall RMSD, while the TM region the lowest (Fig 2). This observation was confirmed by the RMSF analysis (Fig 3), which highlights peaks associated with the large and highly mobile loops.

Cluster analysis was also performed on the three replicas, calculating the distance matrix solely on the more stable transmembrane region. From S5A Fig, very little difference between the replicas can be observed. Only one replica seems to assume different conformations, but S5B Fig in which the medoids of the two observed clusters are superposed and coloured by RMSD, showcases minimal differences.

A thorough analysis of the transport funnel highlighted the presence of several aromatic residues. Among them, three residues close to the intracellular side of the membrane (Y211, F215, and Y445) seem to form a gate-like substructure (S6 Fig).

### Analysis of protein::ligand complexes

Because of the presence of a ligand in the Cryo-EM structure of OCT3, the molecular docking calculation was performed on the minimized model, rather than a representative conformation extracted from the MD simulation, during which the size of the recognition site varied due to the absence of a bound ligand. The Induced-Fit procedure was used to favour the correct orientation of the side chains of the modelled residues and to allow them to better adapt to the ligand. The results of the docking calculations are reported in Table 2.

**Table 2:** Docking score and glide energies of the best pose for each ligand.

Solute	Docking score [kcal/mol]	Glide energy [kcal/mol]
Cytarabine	-10.3	-47.4
Gemcitabine	-10.2	-43.2
Quinidine	-10.1	-34.3
Verapamil	-10.0	-61.7
Fludarabine	-9.6	-38.1
Ipratropium	-9.6	-46.1
Saracatinib	-8.9	-61.9
Tiotropium	-8.2	-45.3
Ribavirin	-7.8	-40.7
Emtricitabine	-7.8	-38.4
Clofarabine	-7.8	-45.6
Phenformin	-7.3	-26.7
Ethambutol	-7.2	-35.0
Amisulpiride	-7.2	-47.2
Entecavir	-6.6	-51.7
Buformin	-5.9	-22.8
Ergothioneine	-5.1	-28.3
Homostachydrine	-4.2	-16.6
Stachydrine	-4.0	-9.8
Carnitine	-3.9	-22.0
Acetylcholine	-3.1	-17.4
Tea	-2.9	-15.3
Metformin	-2.9	-18.8
2-deoxycytidine	-2.5	-42.7
5-fluorouracil	-1.5	-16.5
Choline	-0.5	-20.0
Spermine	-0.9	-31.2
Gabapentin	0.4	-20.9

Interestingly, the top scoring ligands are those known to be the best inhibitors of OCTN1, such as cytarabine and verapamil.

In order to assess the stability of the complexes, as well as the most meaningful interactions between the protein and the small molecule, two replicas of 100 ns MD simulations were run for each complex.

**Fig 4:**
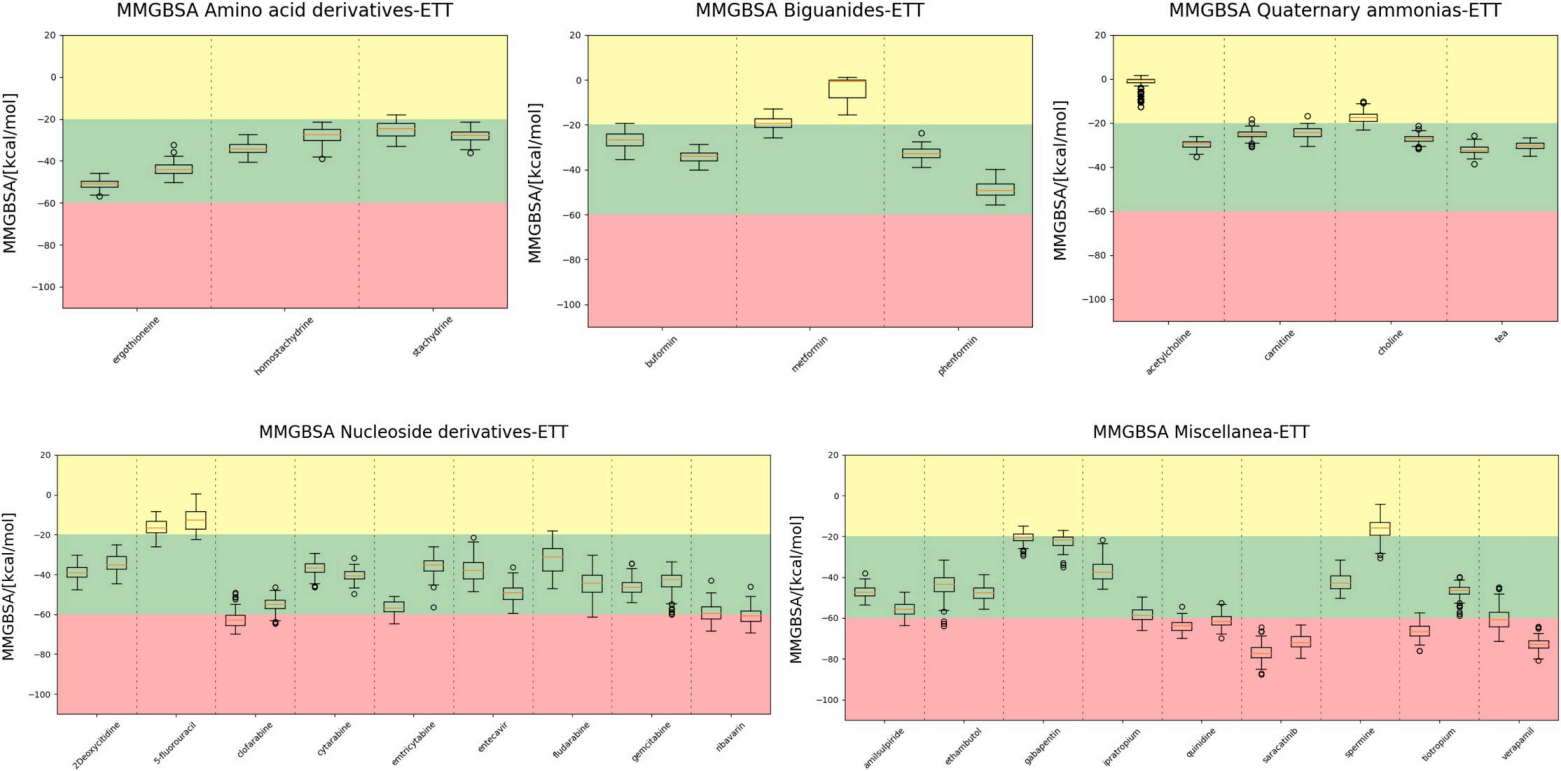
MM/GBSA values between OCNT1 and each ligand during the MD simulations. Two replicas have been run for each complex, therefore there are two boxplots for each solute. Three areas can be identified, in yellow there are weak binders, in red strong binders, with inhibitor-like properties, and in green, good binders.

MM/GBSA values of the protein::solute complexes were calculated in the last 100 frames (10 ns) to assess the binding energy of the complexes (Fig 4). Due to the inherent approximations in these calculations, it is challenging to definitively identify the better solutes for transport when their values are very close, but the MM/GBSA distribution might help in differentiating between three categories of transported solutes:

- Weak Binders (yellow area): Solutes with MM/GBSA values higher than -20 kcal/mol fall into this category. These molecules might be recognized by OCTN1, but their affinity does not appear to be strong. This does not necessarily mean that they are not transported by OCTN1, but simply that they have a low affinity for the outward facing conformation. This data supports the hypothesis that Ach is mostly transported towards the outside of the membrane, and, therefore, a low binding affinity for this conformation should be expected.
- Good Binders (green area): Solutes with MM/GBSA values ranging between -20 and -60 kcal/mol can be categorized as "good binders." Most of the molecules studied in this work belong to this category, including ergothioneine. These solutes exhibit sufficient affinity for the recognition site to remain within the transport funnel but are not overly affine to become trapped in the extracellular part of the funnel.
- Strong Binders (red area): Solutes with MM/GBSA values lower than -60 kcal/mol may be considered "strong binders". These molecules possess an exceptionally high affinity, similar to the behaviour of an inhibitor, rather than a transported solute. Notable examples in this category include quinidine, verapamil, and saracatinib.

**Fig 5:**
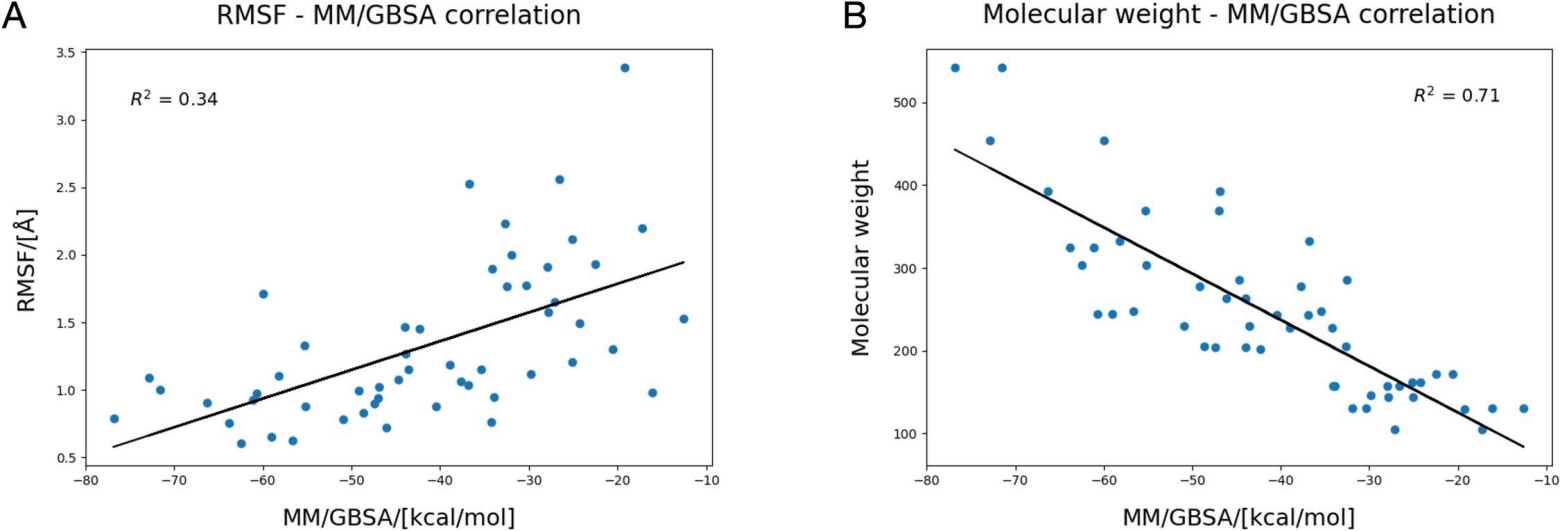
Regression line between the values of MM/GBSA for each ligand and their respective RMSF (A) and molecular weight (B). The coefficient of determination suggests a moderate direct relationship between MM/GBSA and the mobility of the ligand, and a strong inverse relationship between MM/GBSA and the molecular weight of the ligand itself.

Linear regression was also performed between the average MM/GBSA values for each replica, the molecular weight of the solutes, and their average RMSF. In Fig 5 it is possible to see that there is a strong inverse correlation between MM/GBSA and molecular weight (R^2^ = 0.70), while there is a moderate direct correlation between MM/GBSA and RMSF (R^2^ = 0.33). These interesting observations seem to suggest that the molecular weight of the molecules can be a strong indicator of the affinity for the recognition site and, consequently, the transport rate.

Interactions between each solute and OCTN1 were evaluated throughout the replicas in order to identify key transport funnel residues for the recognition and observe if any pattern emerged. In particular, hydrogen bonds, salt bridges, π–π interactions, cation-π interaction, hydrophobic interactions, and water bridges were considered (S7 Fig). The most significant residues forming both hydrogen bonds and salt bridges with many of the docked solutes are Tyr211, Glu381, and Arg469.

Focusing on one of the main transported solutes, ET, it forms interactions with few residues, mainly Tyr211, Phe239, Tyr445, and Arg469. This pattern of interactions can also be seen by the molecules in the same chemical class, namely stachydrine and homostachydrine, particularly regarding Arg469. Additionally, none of these amino acid derivatives interact with Glu381, which most of the other solute interact with, particularly the biguanides buformin and phenformin, as well as ethambutol. Among other interesting observation, it can be observed that ribavirin forms interactions with very few residues, namely Asn32, Gly36, Gln207, Arg469, and Ser472, but these interactions are very well preserved throughout both replicas. Overall, while these data may not allow to detect the presence of clearly distinct binding sites, as hypothesised in literature [3], they help with the identification of some key recognition residues, some of which are common to most solutes.

The RMSF was calculated for the α-carbons of the three most common residues interacting with the small molecules (TYR211, GLU381, and Arg469), reported in S8 Fig. No significant differences could be observed, regardless of the presence of an interaction of those residues with the docked molecule, mostly because all values are relatively low (<1Å). This could be explained by their position in the transmembrane helices, which has an inherently low mobility compared to the rest of the protein. Interestingly, TYR211 was, among the three, the residue with the lowest values in all MD simulations.

The interaction energies between the three key residues and the ligands were also calculated (S9 Fig). In this case as well, not significant differences could be observed between residues of the single simulation, but few exceptions were found. The most noticeable is one of the two spermine replicas, in which interaction energies are much higher compared to the other simulations. This is easily explained by the exit of spermine from the transport funnel. The most peculiar profile is the one obtained by both ethambutol replicas. In fact, despite forming a strong salt bond with Glu381 throughout the simulations, the interaction energy is much higher than the average, with Glu381 as well as with Tyr211 and Arg469. This behaviour might be further explored in future studies employing enhanced sampling molecular dynamics to simulate the binding and unbinding energies.

### Role of sodium in solute recognition

Literature data suggests that sodium is co-transported by OCTN1, particularly, ergothioneine transport increases as sodium concentration increases [41]. Other substrates, such as TEA and Ach, show a decreased transport rate when sodium is present at higher concentrations (7). No data is available regarding the position of the hypothesized Na+ site, but amino acids with a negatively charged side chain (Glu/Asp) should be crucial for the positive ion recognition. However, other residues, such as Tyr, Thr, or Ser, are also likely to participate in Na^+^ binding. Notably, our model predicts that the only Glu/Asp residue present in the transport funnel is Glu381, indicating its potential role in Na+ binding.

Based on the findings from the study on the EEAT and SERT transporters [42, 43] it is plausible to hypothesize a similar sequence of events involving sodium’s role in solute recognition and transport for OCTN1. Initially, the Na+ binding pocket opens, allowing for the influx of water molecules. Subsequently, a Na+ ion enters the pocket and interacts with the pocket residues. The binding triggers conformational changes that facilitate the formation of the solute recognition site.

Obviously, because the model is already in the open conformation, the dynamic process of water molecules filling the site could not be simulated using classical techniques, as it is already fully solvated and ready for the solute binding. Therefore, this part of the study focused on the analysis of the Na^+^ molecules paths during the simulations. In order to observe how different ion concentrations can affect its entrance in the transport funnel, aside from the three equilibration replicas at 150mM of Na^+^/Cl^-^, three 1 μs MD replicas at 400mM and 1000mM were also performed, and the sodium densities in the simulation boxes analyzed. In S10 Fig a higher occupancy in the transport funnel can be identified around the residue Glu381, as previously hypothesized, strongly implying its role in Na^+^ binding. Additionally, there appears to be a clear correlation between Na^+^ concentrations and Na^+^ density in the transport funnel. This relationship supports the experimental observation that sodium concentration has a direct effect on solute transport (mostly ergothioneine), as it has a higher probability of entering the transport funnel and, therefore, starting the transport process.

**Fig 6:**
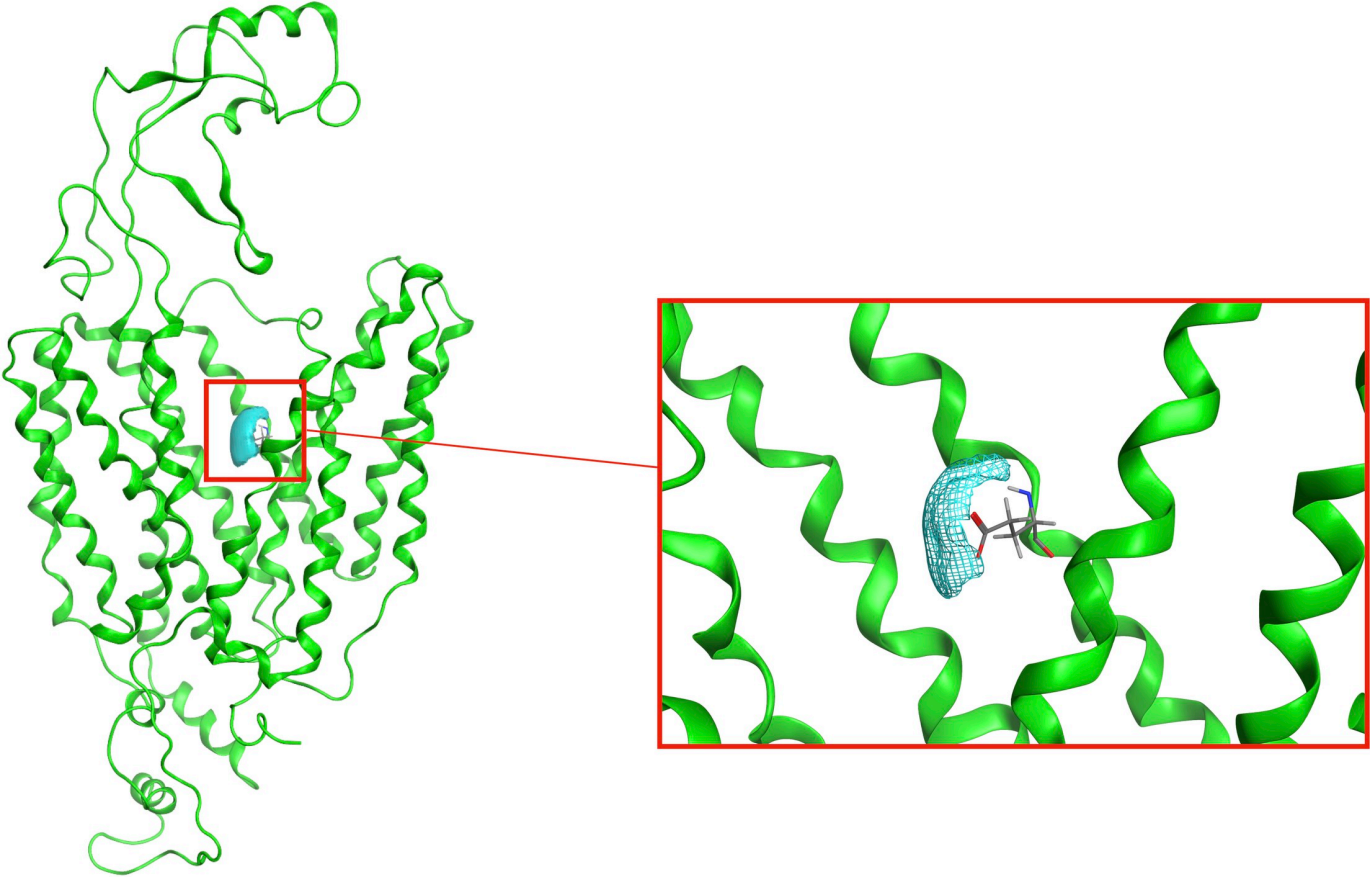
Na+ ion density region inside the transport funnel obtained by MOE’s 3D-RISM. The extracellular portion is in the upper side of the figure.

This putative recognition site is further supported by the results of the RISM-3D analysis, used to compute the density functions of Na^+^ ions nearby residues forming the transport funnel. In Fig 6 one region was clearly identified as a putative site for sodium binding, formed by the negative charge of the side chain of Glu381.

**Fig 7:**
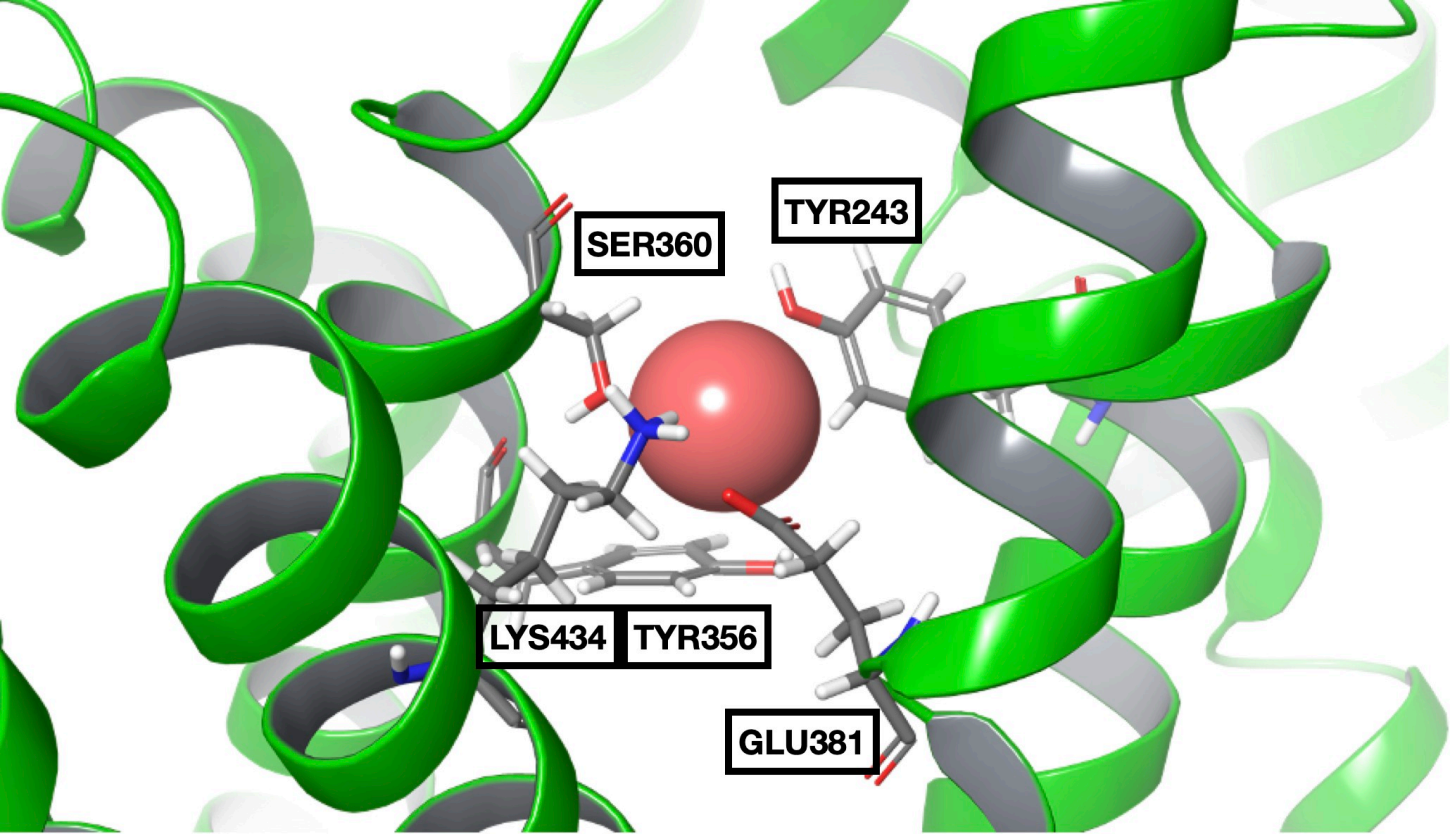
Frame extracted from the OCNT1::Na+ MD simulation. The sodium ion can be seen interacting with Tyr243, Ser360, Tyr356, Glu381, and Lys434.

One Na^+^ ion was then placed in the coordinates identified by that region and the nearby residues were minimized. Then, this minimized complex was used as a starting point for a 100 ns MD simulation to assess the behaviour of the Na+ ion inside the transport funnel if already placed there. It can be observed that the residues surrounding the ion initially rearrange themselves, increasing the number of interactions with Na+ (Fig 7). However, after ∼45 μs, the interactions are lost, and the ion exited the transport funnel.

It is possible to hypothesize that the ion binds for a sufficient duration to facilitate the recognition of the solute. However, the forces keeping the solute interacting with the protein are not strong enough to keep it there for a long time. Therefore, if the concentration of either the solute or Na^+^ is too low, the time the ion spends in the binding site will not be enough to facilitate solute recognition and transport.

Interaction energies between the ion and Glu381 were calculated (S11 Fig), and the frame with the lowest value was selected and extracted to serve as the receptor for subsequent molecular docking. From Fig 7 several residues can be observed, close enough to interact with the Na+ ion in the extracted frame, in particular Tyr243, Ser360, Tyr356, Glu381, and Lys434.

**Fig 8:**
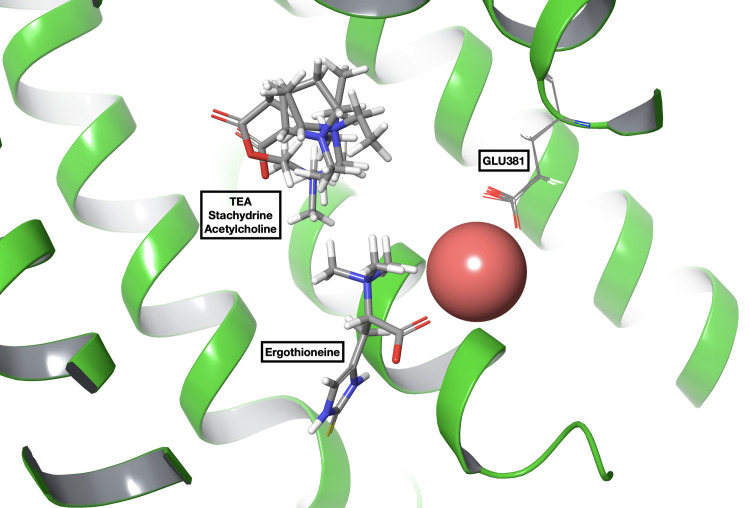
Best pose obtained by the docking procedure of ergothioneine, TEA, stachydrine and Ach using the OCNT1:Na^+^ complex as receptor. While ergothioneine can be seen interacting with Na+, the three other solutes are unaffected by its presence.

Induced-fit molecular docking of the solutes was conducted in the presence of sodium in the described site to observe the differential influence of the Na+ ion on their poses, juxtaposed to the first round of docking calculations, in which no sodium ion was present. Intriguingly, as depicted in Fig 8, ergothioneine appears to benefit from the ion’s presence through interactions with its negatively charged functional group. In contrast, the poses of other solutes, like TEA, stachydrine, and Ach remain unaffected by the cation. These findings shed light on the specific role of Na+ in substrate recognition and transport. Its presence in the transport funnel potentially stabilizes solutes with specific physicochemical characteristics, such as ergothioneine, while exerting no effect or even a negative impact on the recognition of other solutes, such as TEA or Ach. Further studies utilizing enhanced sampling techniques to simulate the whole transport mechanism will provide deeper insights into the specific dynamics of sodium co-transport and its overall role in OCTN1 function.

## Conclusions

Based on the results obtained in this study, several key findings can be highlighted. Firstly, a reliable 3D model of the OCTN1 protein was successfully generated through a homology modelling approach, using OCT3 as a template. Despite challenges in aligning the EL1 region, a chimeric model combining the homology model and an AF-generated model was employed to improve the overall model quality. Molecular dynamics simulations provided a deeper understanding of the stability and mobility of the different domains of this transporter.

The analysis of protein::ligand complexes using molecular docking revealed valuable insights. Notably, ligands such as cytarabine and verapamil, known inhibitors of ergothioneine transport, displayed high docking scores, indicating favorable binding. Molecular dynamics simulations of the complexes demonstrated the stability of most ligands within the binding pocket and helped in the identification of the key interaction sites. In particular, Tyr211, Glu381, and Arg469 were found to be significant residues forming multiple hydrogen bonds and salt bridges with the docked solutes. The low interaction energy and low number of interactions between OCTN1 and Ach support the hypothesis that Ach is transported only towards the extracellular compartment. Additionally, relationships between molecular weight of the solute, its mobility in the transport funnel, and affinity for OCNT1 emerged.

Furthermore, the role of sodium in solute transport was investigated. Density function calculations and molecular dynamics at increasing salt concentrations allowed the identification of a putative sodium binding site near Glu381. During the simulations, a dynamic association and dissociation of the ions with the residues in the transport funnel could be observed. The impact of Na+ on solute recognition was assessed via further molecular docking calculations, showing that its presence in the transport funnel improved ergothioneine recognition, while not affecting other solutes.

In conclusion, this study provides important insights into the structure, dynamics, and ligand interactions of OCNT1. The generated 3D model and characterization of key residues involved in ligand recognition pave the way for further investigations and potential applications in drug discovery. The role of sodium in ergothioneine transport highlights the complex interplay between ions and solutes, contributing to our understanding of the transport mechanisms in this protein. Overall, the data provided in this study will positively contribute to the discussion regarding this still controversial transporter, specifically regarding the recognition site of Na+ and its role in solute transport.

In the future, further investigations can focus on the impact of mutations in OCNT1 on ligand recognition and transport. The whole transport mechanism and the role of Na+ in this process could be also studied by enhanced-sampling MD simulations. Additionally, experimental validation of the predicted interactions and binding modes using techniques like site-directed mutagenesis and functional assays would strengthen the understanding of OCNT1’s mechanism and aid in the development of novel therapeutic strategies, specifically, new inhibitors to be used in cancer therapy.

## Supporting information

S1 Fig Sequence alignment of OCNT1 and OCT3. Residues are coloured in a scale from red to blue at increasing levels of similarity. EL1 (residues 42–141), is clearly the part with the lowest overall similarity, therefore OCT3 could not be considered a reliable template to obtain a complete model via homology modelling.
(TIF)

S2 Fig EL1 of 7ZH6 (A) and the AF generated SLC22A4 model (B). In (A) breaks in the structure are clearly visible, a common occurrence when experimentally solving highly unstructured protein portions. The EL1 in (B) is much more structured than in the 7ZH6 structure, but some common features can be observed. In (C) the two loops are superposed. Differences can be clearly observed, particularly in the secondary structure features. In (D) the EL1 of the newly generated AF model and the model deposited in the AF database are compared. No significant differences can be observed, in the conformation, nor in the secondary structure. In all panels, the extracellular portion is in the upper side of the figure.
(TIF)

S3 Fig Predicted IDDT plot of the AF model. The average reliability score is relatively high, the peaks represent highly structured regions of the transporters, such as the transmembrane helices. The trough identified around residues 310–320 is indicative of a highly unstructured portion of the intracellular loop 4 (IL4), which is highly mobile, and, therefore, returns a low plDDT. The portion of interest for this study is predicted with a high level of confidence, except a small turn connecting two structured portions around residues 75–85.
(TIF)

S4 Fig Superposition of the transmembrane helices of the model obtained by homology model (green) and the AF-generated model (blue), as seen from the extracellular compartment. It is evident how the AF model opening towards the outside of the membrane is much more closed as compared to the homology model, which is clearly in the outward open conformation.
(TIF)

S5 Fig A) Clusters obtained from the cluster analysis of the TM region in the three equilibration MDs. B) Medoids of the two obtained clusters, superposed and coloured by RMSD. It is clear that, despite belonging to two different clusters, the transmembrane portions of the two structures are very similar. The extracellular portion is in the upper side of the figure.
(TIF)

S6 Fig Focus on aromatic residues in the model transport funnel. Y211, F215, and F445 seem to work together forming a gate, regulating the access to and from the intracellular side of the membrane when in the outward-facing conformation. The extracellular portion is in the upper side of the figure.
(TIF)

S7 Fig Solute-residue interactions in the last 100 frames of MD replicas. A) Hydrogen bonds, B) Salt bridges, C) π-π interactions, D) Cation-π interactions, E) Hydrophobic interactions, F) Water bridges.
(PDF)

S8 Fig RMSF of TYR211, GLU381, and ARG469 in the last 100ns of MD replicas. The RMSF values were calculated for the α-carbon of each of the three key residues. No significant differences can be observed, as all values range from ∼0.3 Å to ∼1 Å except for very few exceptions. Interestingly, TYR211 is always the residue with the lowest mobility.
(TIF)

S9 Fig Interaction energies of TYR211, GLU381, and ARG469 in the last 100ns of MD replicas. The interaction energies were calculated between the molecules and each of the three key residues. All key residues seem to have a similar interaction energy with each molecule. Notable exceptions are one of the spermine replicas (which can be explained by the ligand exiting the transport funnel) and the two ethambutol replicas.
(TIF)

S10 Fig Density map of the Na+ ions during the MD simulations at A) 150mM, B) 400mM, and C) 1000mM. It is possible to see how, with the increase of sodium concentration, the density of sodium in the transport funnel, close to Glu381, increases in comparison to the average density in the system. Other high-density areas correspond to volumes close to negatively charged residues. The extracellular portion is in the upper side of the figure.
(TIF)

S11 Fig Interaction energies between the Na^+^ placed in the transport funnel and Glu381. It is easy to observe the moment the interaction between the ion and the residue are lost, and the Na^+^ leaves the transport funnel (time ≈ 47ns).
(TIF)
