# Correction: Atomistic description of the OCTN1 recognition mechanism via in silico methods

**DOI:** 10.1371/journal.pone.0315861

**Published:** 2024-12-11

**Authors:** The PLOS ONE Staff

The funding statement for this paper is incorrect. The publisher apologizes for this error.

The correct funding statement is as follows: This research was supported by MUR–Bando PRIN 2022. Omar Ben Mariem, Luca Palazzolo, Yao Wei, Davide Bianchi, Uliano Guerrini, Tommaso Laurenzi, Simona Saporiti, Emma De Fabiani, and Ivano Eberini were supported by grants from MIUR—Progetto Eccellenza 2023–2027. Luca Palazzolo was supported by PSR 2022 –Azione A. This work was also supported by the PRIN 2022 (Progetti di Ricerca di Interesse Nazionale) project code 2022JWT5XS, awarded to Lorena Pochini and Ivano Eberini, and granted by MUR (Ministry of University and Research)–Italy. Additionally, this project has received funding from the European Union’s Horizon Europe research and innovation programme under the Marie Skłodowska-Curie grant agreement No. 101073546 (MSCA Doctoral Network Metal-containing Radical Enzymes–MetRaZymes).

## References

[1] Ben Mariem O, Palazzolo L, Torre B, Wei Y, Bianchi D, Guerrini U, et al. (2024) Atomistic description of the OCTN1 recognition mechanism via in silico methods. PLoS ONE 19(6): e0304512. 10.1371/journal.pone.0304512 38829838 PMC11146731

