# Supplementary figures and images for: Atomistic description of the OCTN1 recognition mechanism via in silico methods
Source: PLoS One. 2024 Jun 3;19(6):e0304512. doi: 10.1371/journal.pone.0304512 (PMC11146731; doi:10.1371/journal.pone.0304512)

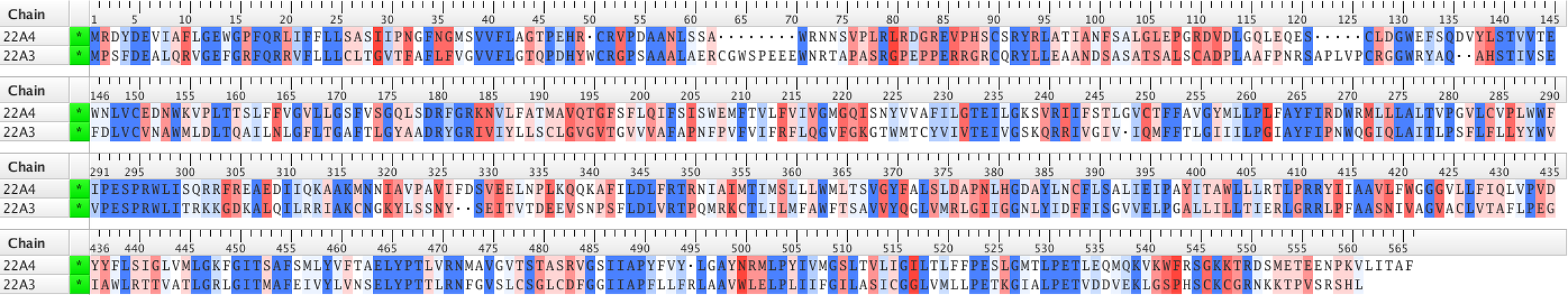

Supplement: S1 Fig — Residues are coloured in a scale from red to blue at increasing levels of similarity. EL1 (residues 42–141), is clearly the part with the lowest overall similarity, therefore OCT3 could not be considered a reliable template to obtain a complete model via homology modelling. (TIF) [file pone.0304512.s001.tif]

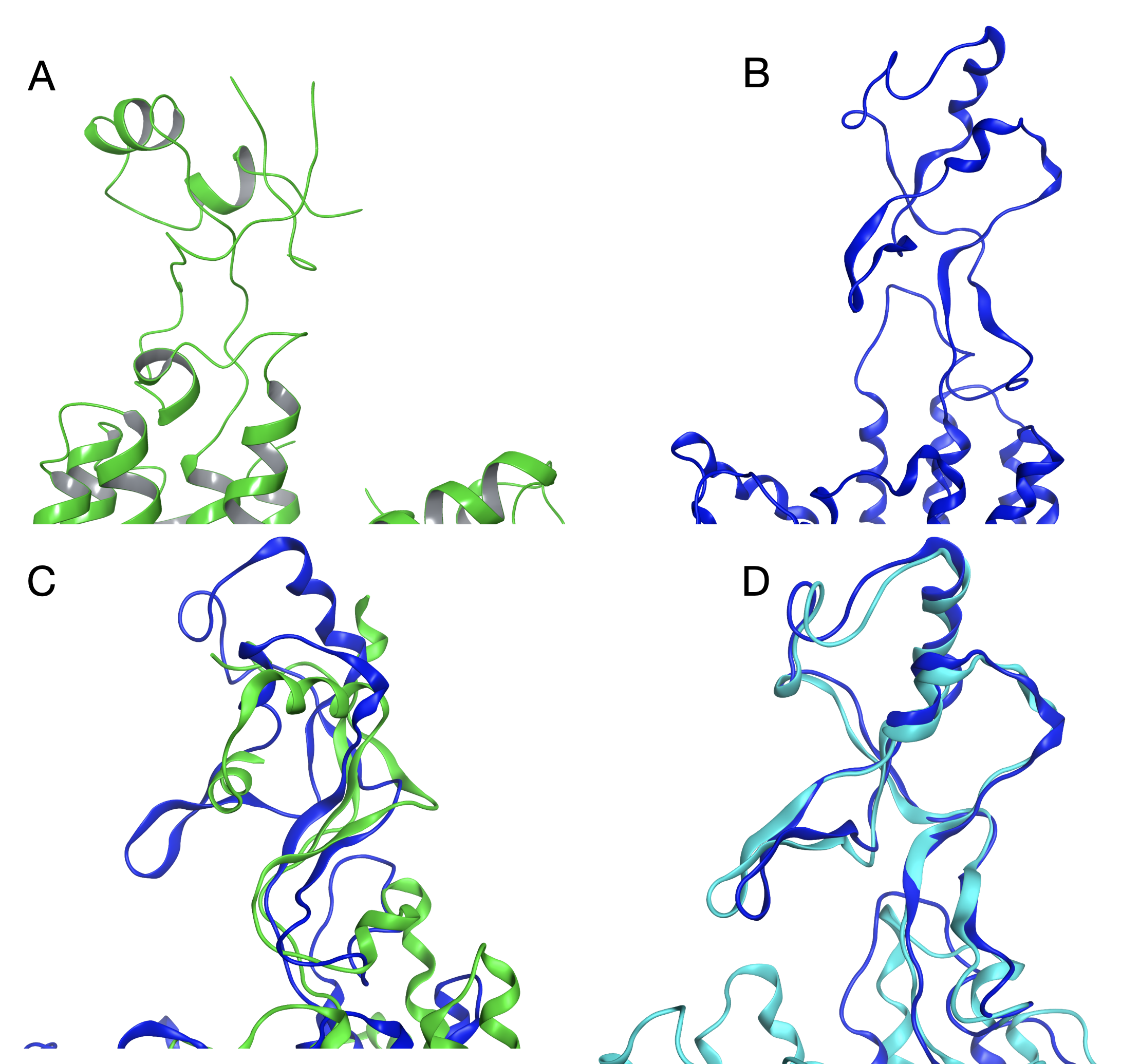

Supplement: S2 Fig — EL1 of 7ZH6 (A) and the AF generated SLC22A4 model (B). In (A) breaks in the structure are clearly visible, a common occurrence when experimentally solving highly unstructured protein portions. The EL1 in (B) is much more structured than in the 7ZH6 structure, but some common features can be observed. In (C) the two loops are superposed. Differences can be clearly observed, particularly in the secondary structure features. In (D) the EL1 of the newly generated AF model and the model deposited in the AF database are compared. No significant differences can be observed, in the conformation, nor in the secondary structure. In all panels, the extracellular portion is in the upper side of the figure. (TIF) [file pone.0304512.s002.tif]

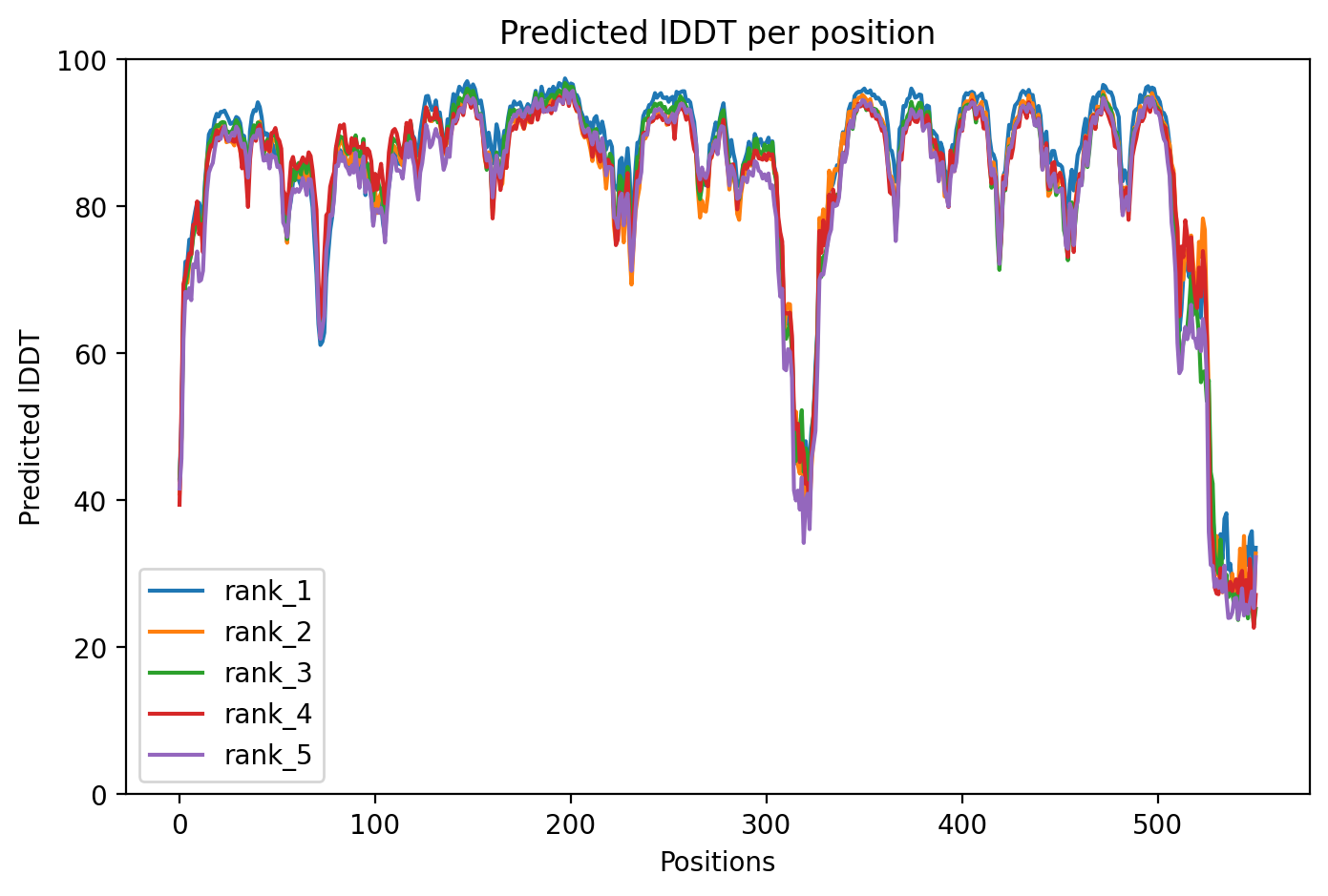

Supplement: S3 Fig — The average reliability score is relatively high, the peaks represent highly structured regions of the transporters, such as the transmembrane helices. The trough identified around residues 310–320 is indicative of a highly unstructured portion of the intracellular loop 4 (IL4), which is highly mobile, and, therefore, returns a low plDDT. The portion of interest for this study is predicted with a high level of confidence, except a small turn connecting two structured portions around residues 75–85. (TIF) [file pone.0304512.s003.tif]

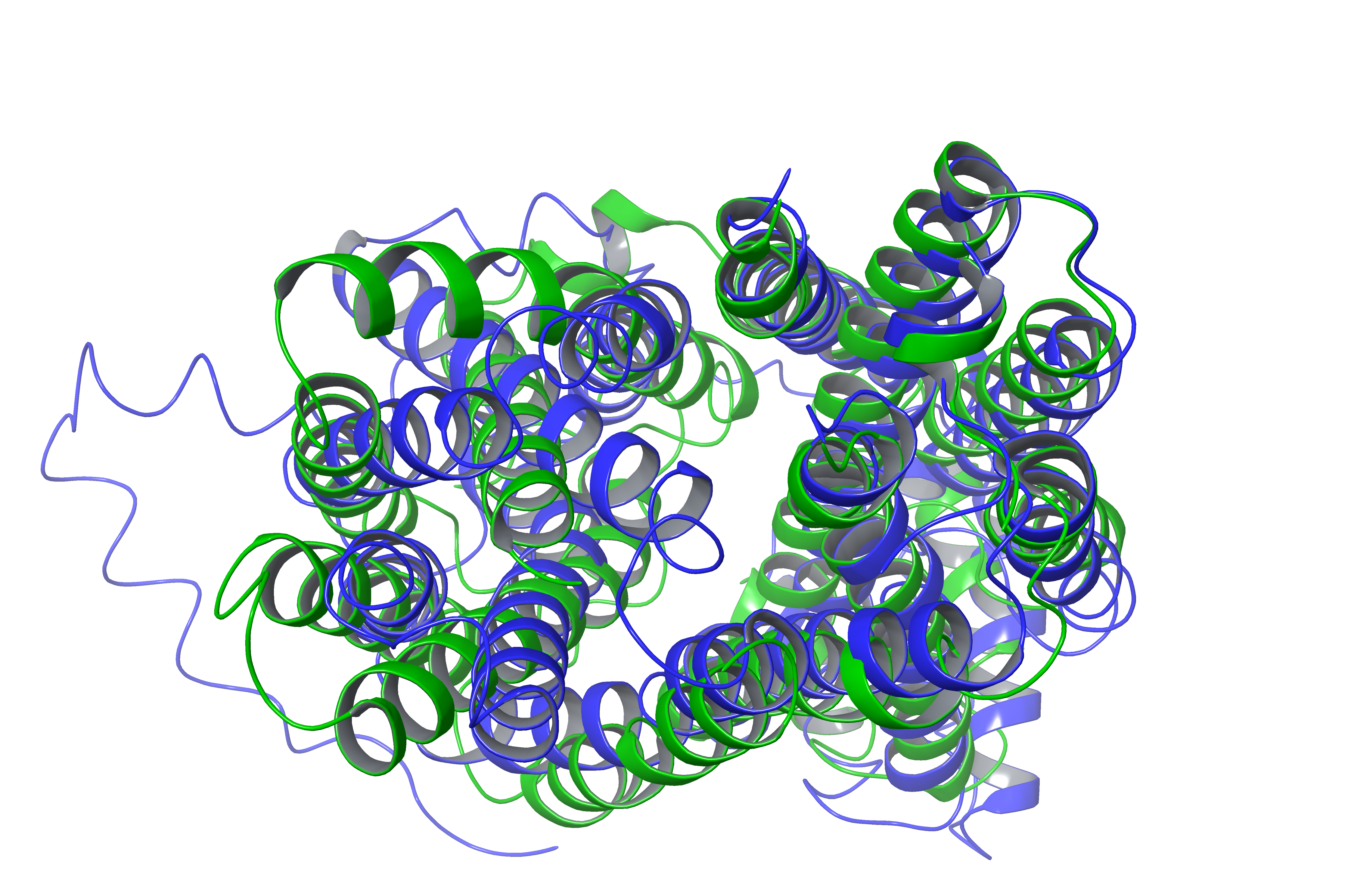

Supplement: S4 Fig — It is evident how the AF model opening towards the outside of the membrane is much more closed as compared to the homology model, which is clearly in the outward open conformation. (TIF) [file pone.0304512.s004.tif]

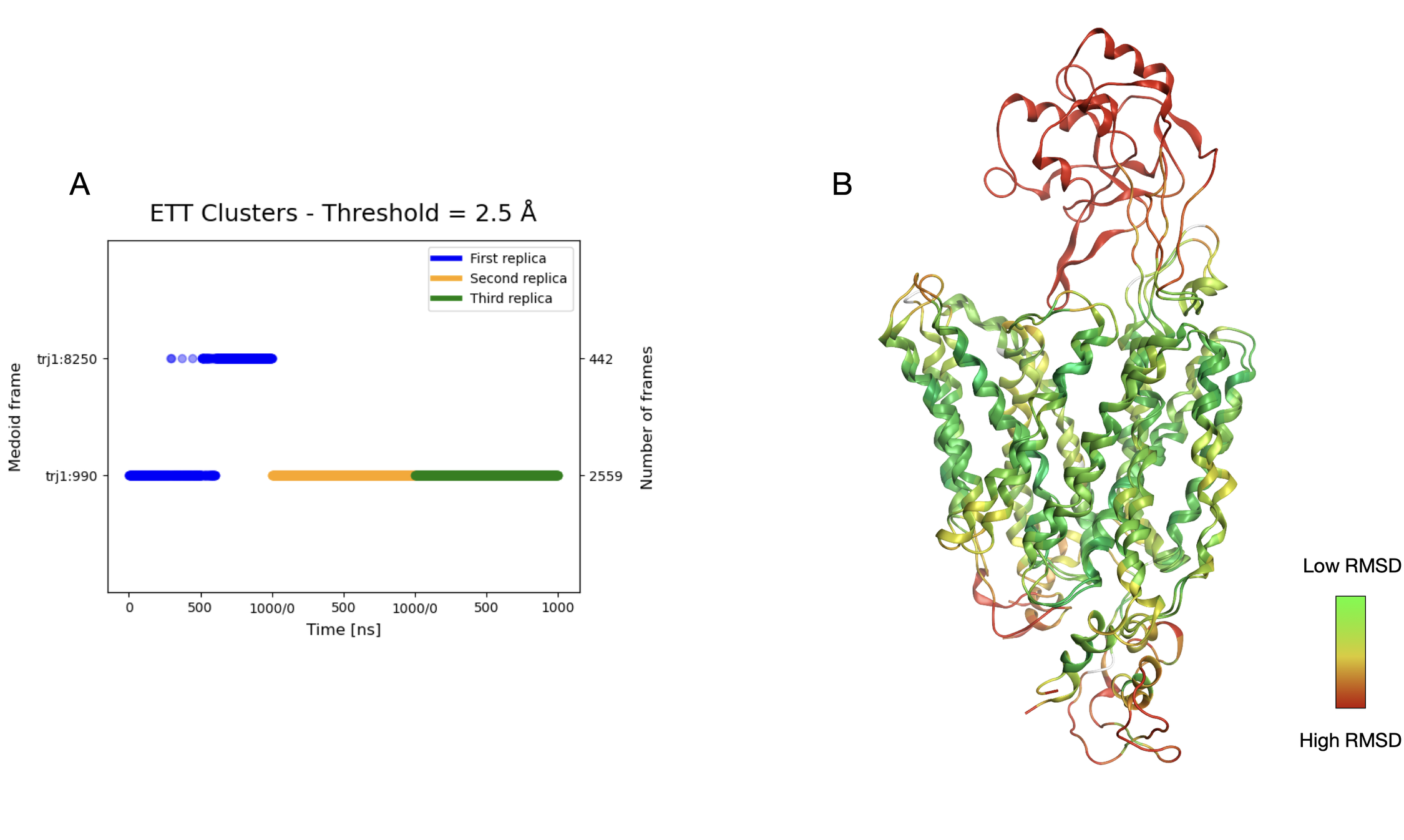

Supplement: S5 Fig — A) Clusters obtained from the cluster analysis of the TM region in the three equilibration MDs. B) Medoids of the two obtained clusters, superposed and coloured by RMSD. It is clear that, despite belonging to two different clusters, the transmembrane portions of the two structures are very similar. The extracellular portion is in the upper side of the figure. (TIF) [file pone.0304512.s005.tif]

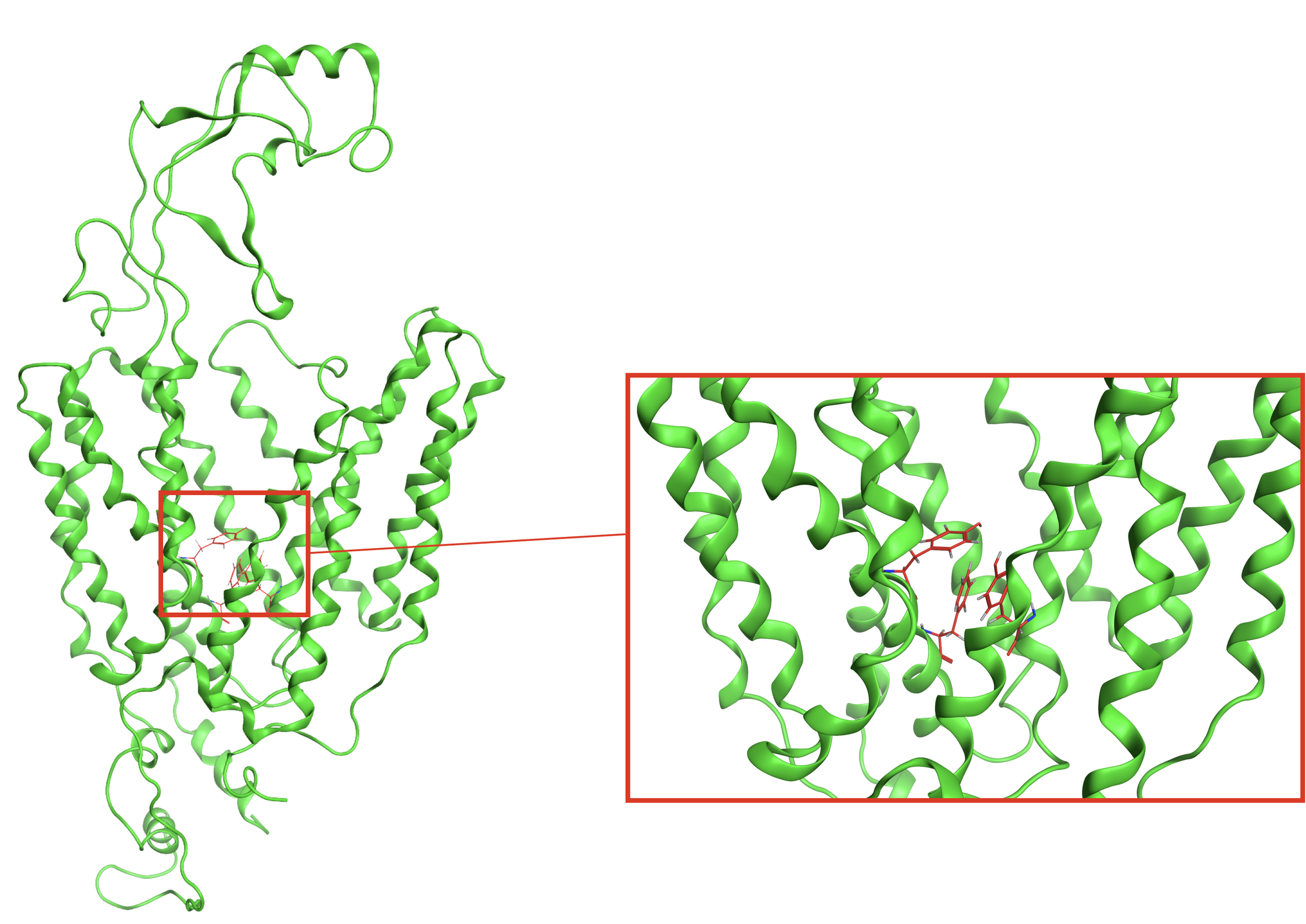

Supplement: S6 Fig — Y211, F215, and F445 seem to work together forming a gate, regulating the access to and from the intracellular side of the membrane when in the outward-facing conformation. The extracellular portion is in the upper side of the figure. (TIF) [file pone.0304512.s006.tif]

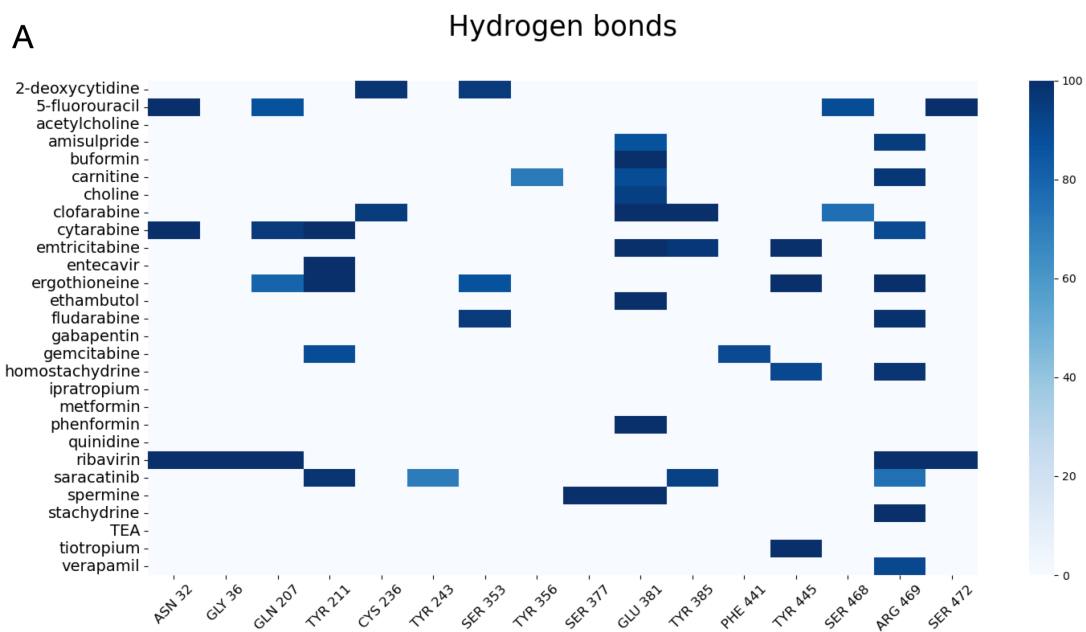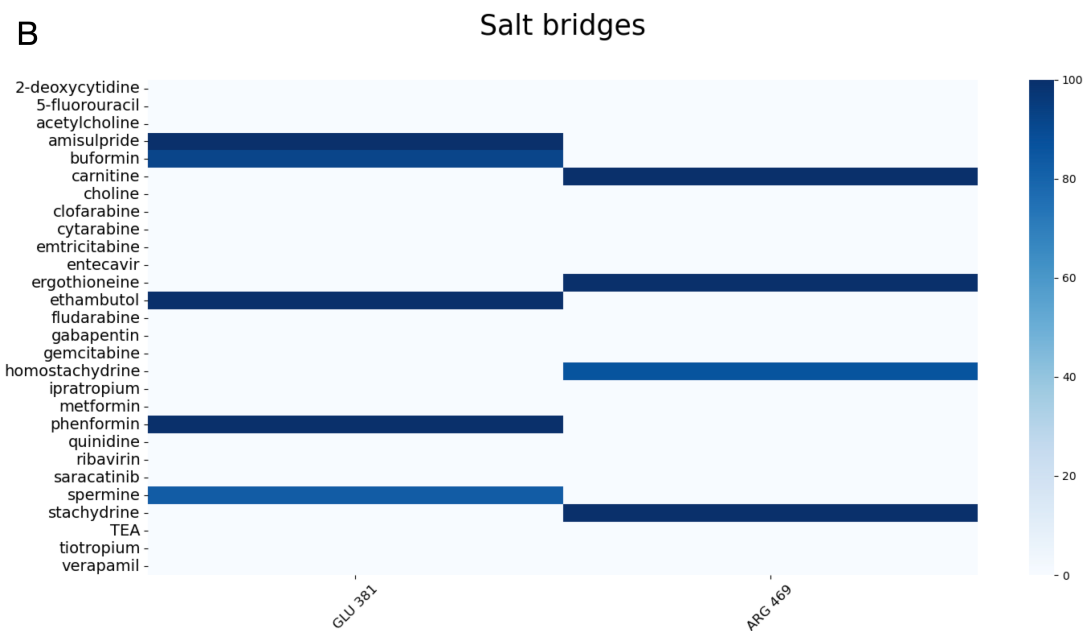

## C $\pi$ - $\pi$ interactions

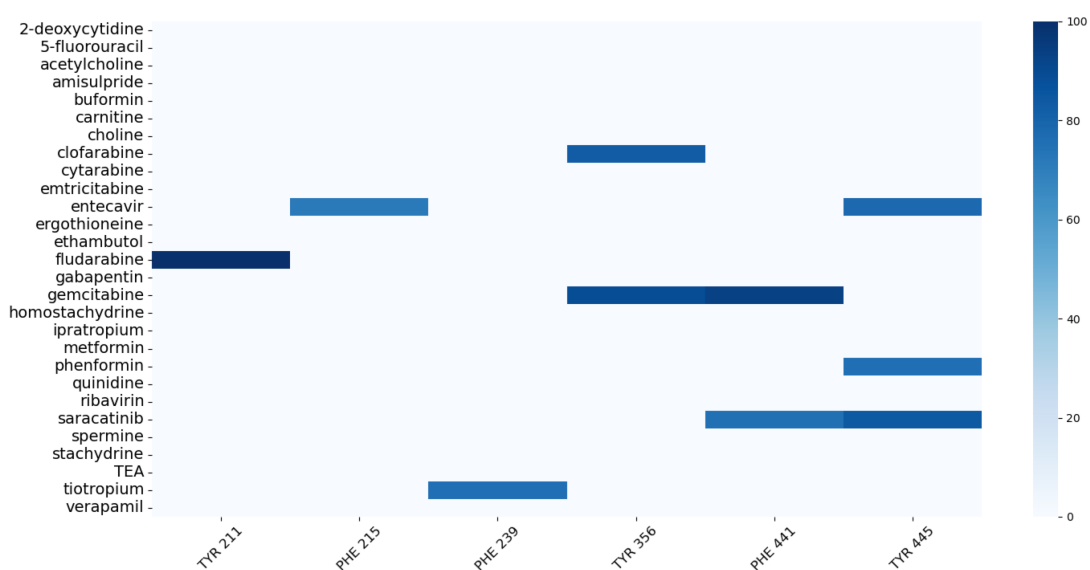

## D Cation- $\pi$ interactions

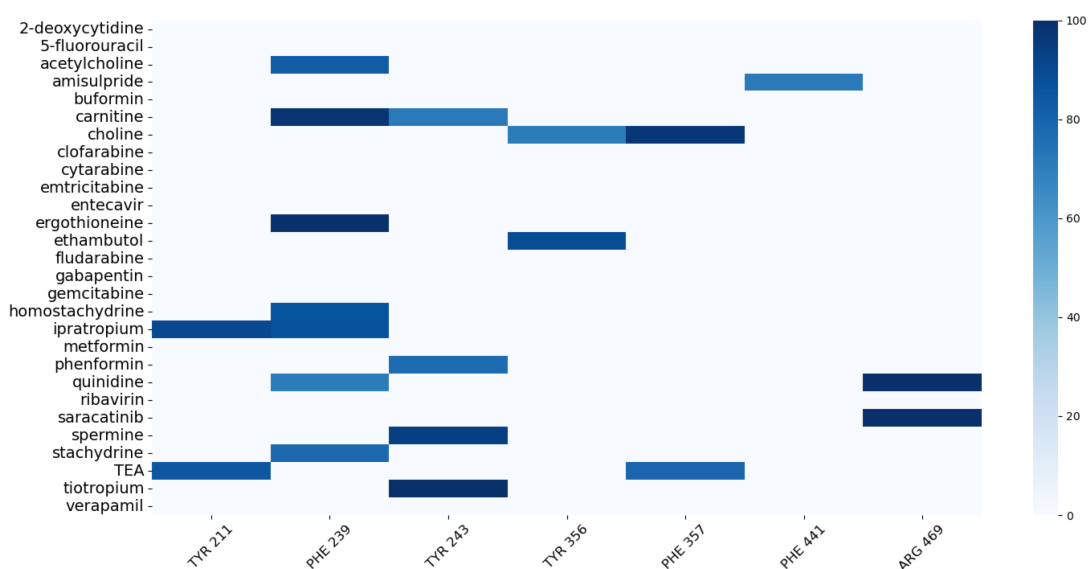

## E Hydrophobic interactions

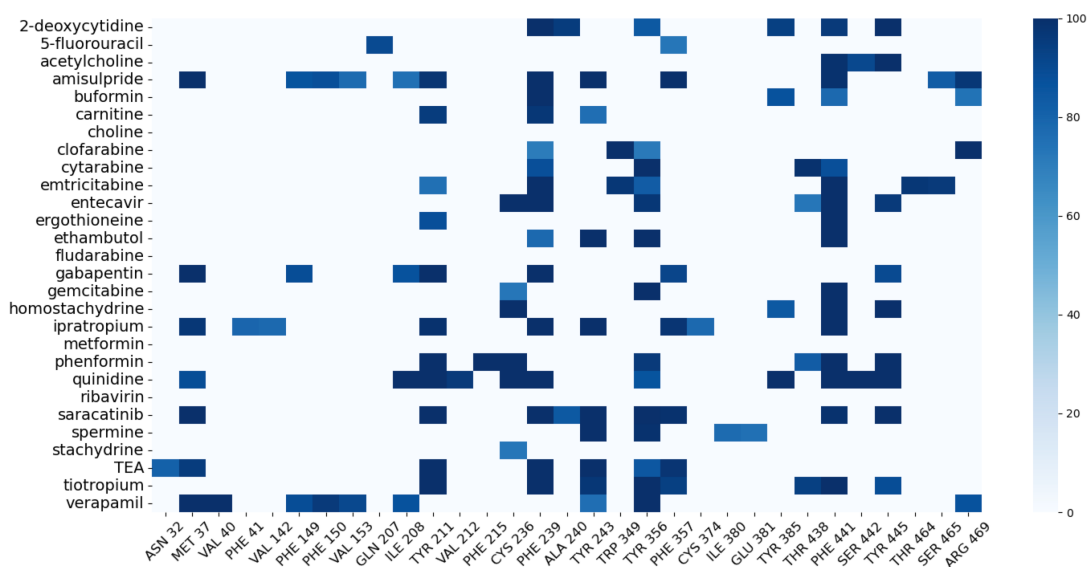

## F Water bridges

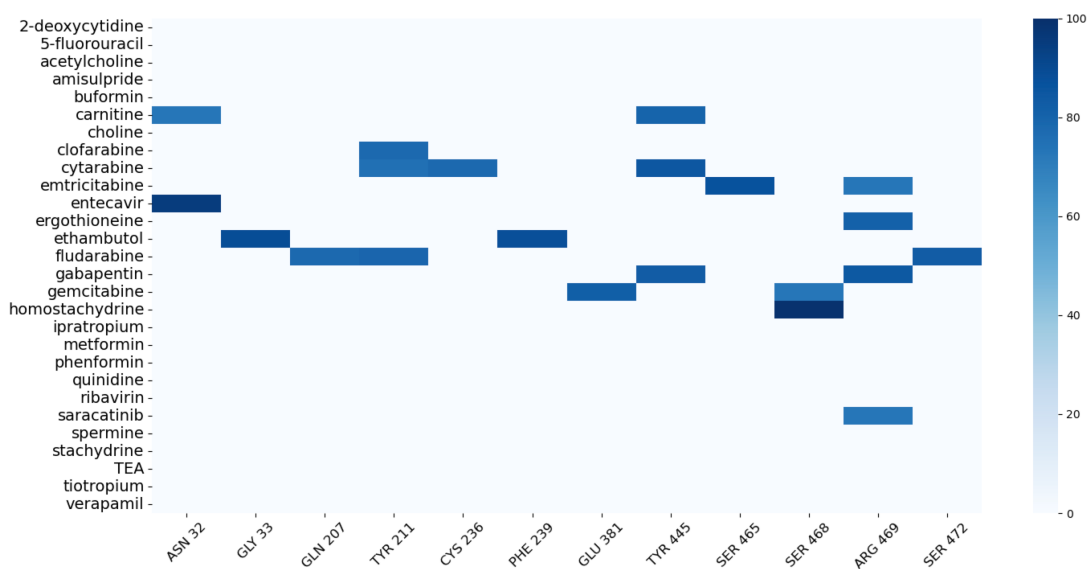

Supplement: S7 Fig — A) Hydrogen bonds, B) Salt bridges, C) π-π interactions, D) Cation-π interactions, E) Hydrophobic interactions, F) Water bridges. (PDF) [file pone.0304512.s007.pdf]

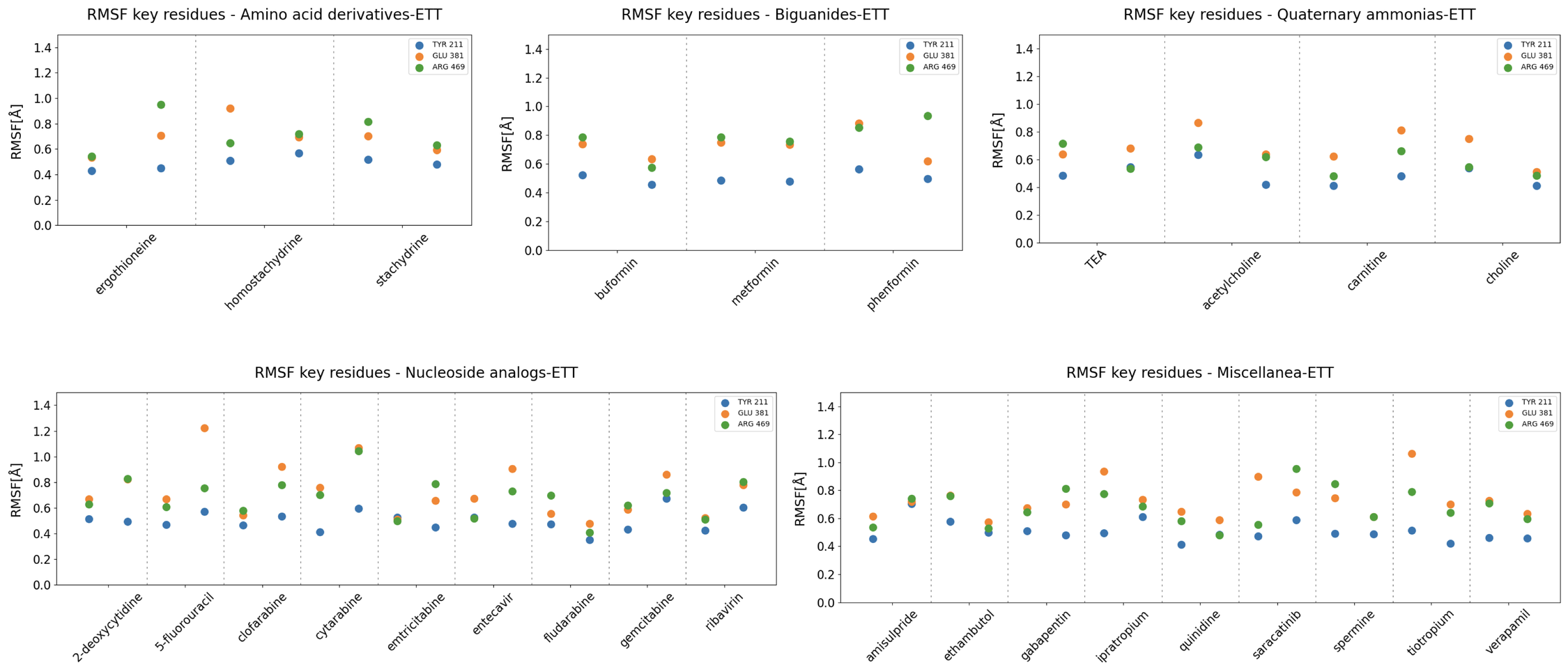

Supplement: S8 Fig — The RMSF values were calculated for the α-carbon of each of the three key residues. No significant differences can be observed, as all values range from ∼0.3 Å to ∼1 Å except for very few exceptions. Interestingly, TYR211 is always the residue with the lowest mobility. (TIF) [file pone.0304512.s008.tif]

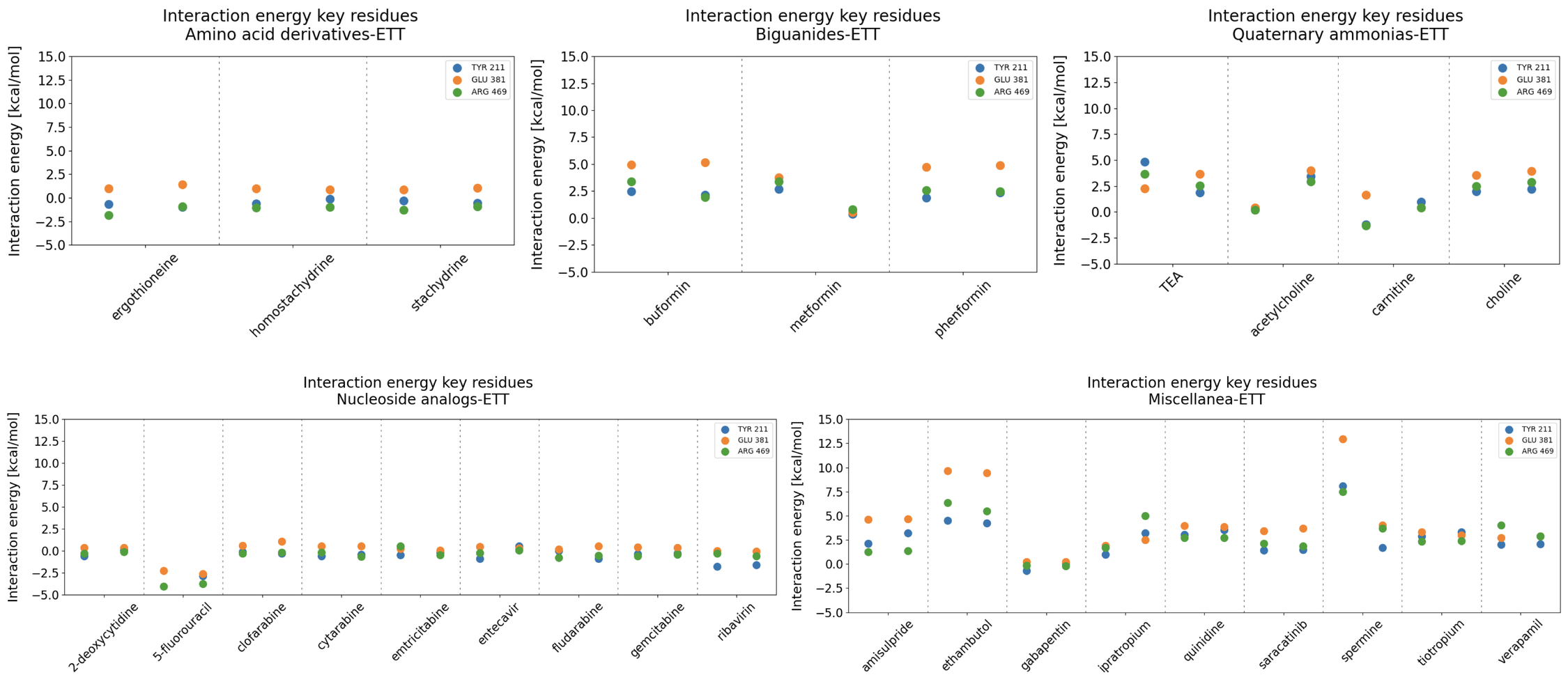

Supplement: S9 Fig — The interaction energies were calculated between the molecules and each of the three key residues. All key residues seem to have a similar interaction energy with each molecule. Notable exceptions are one of the spermine replicas (which can be explained by the ligand exiting the transport funnel) and the two ethambutol replicas. (TIF) [file pone.0304512.s009.tif]

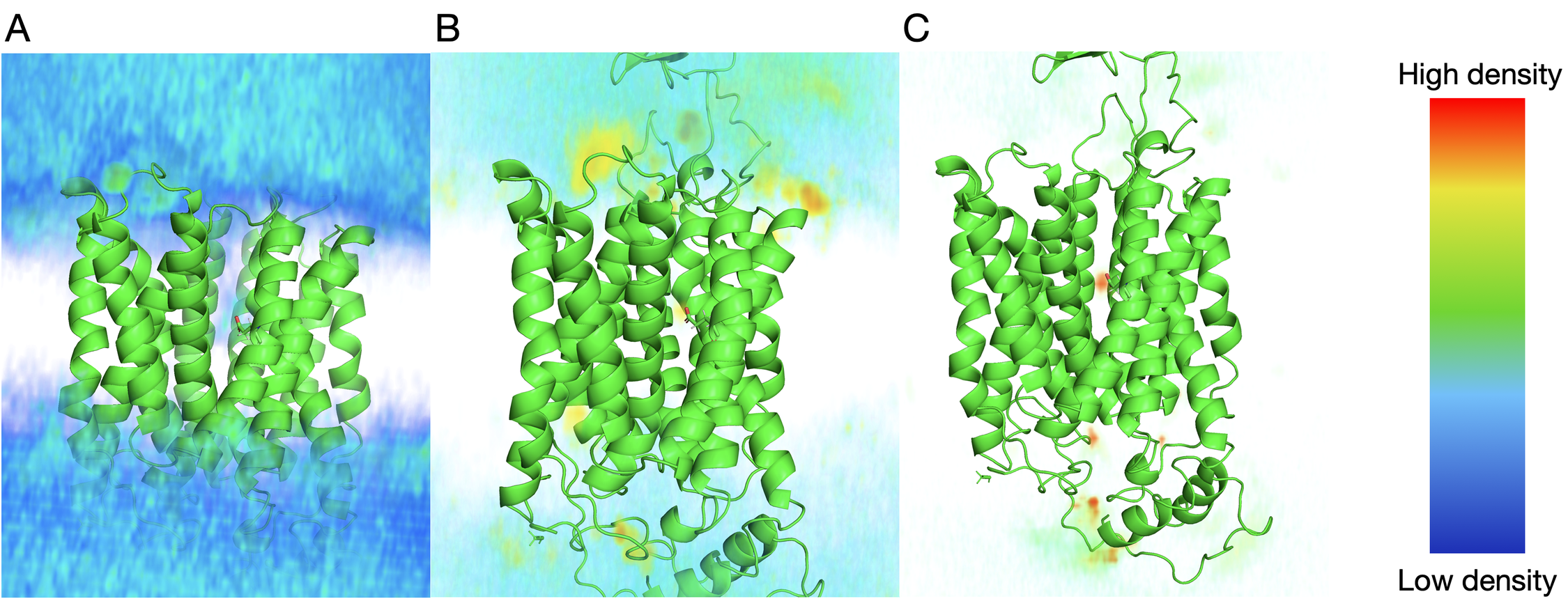

Supplement: S10 Fig — Density map of the Na+ ions during the MD simulations at A) 150mM, B) 400mM, and C) 1000mM. It is possible to see how, with the increase of sodium concentration, the density of sodium in the transport funnel, close to Glu381, increases in comparison to the average density in the system. Other high-density areas correspond to volumes close to negatively charged residues. The extracellular portion is in the upper side of the figure. (TIF) [file pone.0304512.s010.tif]

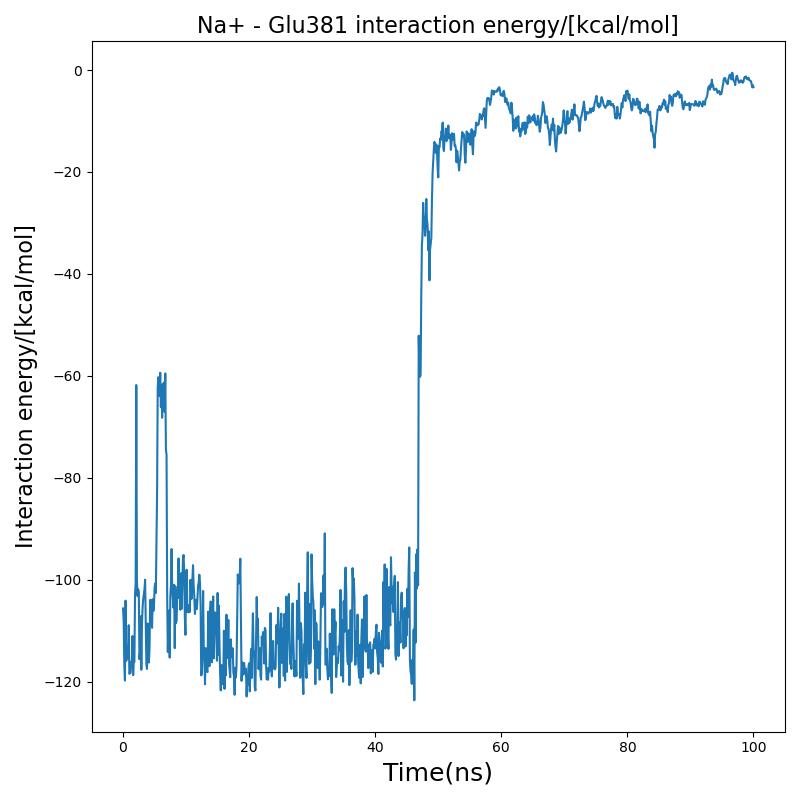

Supplement: S11 Fig — It is easy to observe the moment the interaction between the ion and the residue are lost, and the Na+ leaves the transport funnel (time ≈ 47ns). (TIF) [file pone.0304512.s011.tif]
